# Discovery of Non-Covalent Inhibitors for SARS-CoV-2 PLpro: Integrating Virtual Screening, Synthesis, and Experimental Validation

**DOI:** 10.1021/acsmedchemlett.4c00420

**Published:** 2024-12-02

**Authors:** Bruna K. P. Sousa, Melina Mottin, Donald Seanego, Christopher D. Jurisch, Beatriz S. A. Rodrigues, Verônica L. S. da Silva, Milene Aparecida Andrade, Gilberto S. Morais, Diogo F. Boerin, Thamires Q. Froes, Flávia Nader Motta, M. Cristina Nonato, Izabela D. M. Bastos, Kelly Chibale, Richard K. Gessner, Carolina Horta Andrade

**Affiliations:** † Center for the Research and Advancement in Fragments and Molecular Targets (CRAFT), Faculdade de Ciências Farmaceuticas de Ribeirão Preto, Universidade de São Paulo, Ribeirão Preto, São Paulo 05508-070, Brazil; ‡ Laboratory for Molecular Modeling and Drug Design (LabMol), Faculdade de Farmácia, Universidade Federal de Goiás, Goiânia, Goiás 74690-900, Brazil; § Holistic Drug Discovery and Development Centre (H3D), University of Cape Town, Cape Town 7701, South Africa; ∥ Pathogen-Host Interface Laboratory, Department of Cell Biology, University of Brasilia, Brasilia 73345-010, Brazil; ⊥ Laboratório de Cristalografia de Proteínas, Faculdade de Ciências Farmacêuticas de Ribeirão Preto, Universidade de São Paulo, Ribeirão Preto, São Paulo 05508-070, Brazil; # Faculdade de Ceilândia, Universidade de Brasília, Brasília, Distrito Federal 73345-010, Brazil; ∇ Center for Excellence in Artificial Intelligence (CEIA), Instituto de Informática, Universidade Federal de Goiás, Goiânia, Goiás 74690-900, Brazil; ○ South African Medical Research Council Drug Discovery and Development Research Unit, University of Cape Town, Cape Town 7701, South Africa; ⧫ Institute of Infectious Disease and Molecular Medicine, University of Cape Town, Cape Town 7701, South Africa

**Keywords:** SARS-CoV-2, COVID-19, papain-like protease (PLpro), virtual screening, synthesis, enzymatic assay, noncovalent inhibitor, naphthyridine

## Abstract

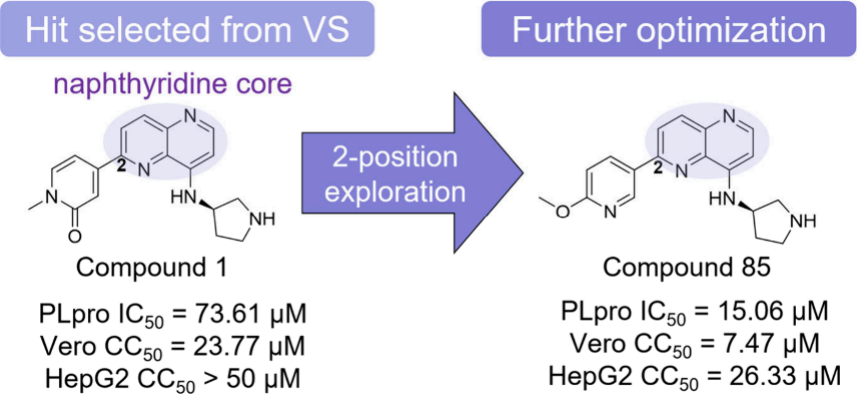

The SARS-CoV-2 pandemic has significantly challenged global public health, highlighting the need for effective therapeutic options. This study focuses on the papain-like protease (PLpro) of SARS-CoV-2, which is a critical enzyme for viral polyprotein processing, maturation, and immune evasion. We employed a combined approach that began with computational models in a virtual screening campaign, prioritizing compounds from our in-house chemical library against PLpro. Out of 81 virtual hits evaluated through enzymatic and biophysical assays, we identified a modest inhibitor featuring a naphthyridine core with an IC_50_ of 73.61 μM and a *K*_i_ of 22 μM. Expanding our exploration, we synthesized and assessed 30 naphthyridine analogues, three of which emerged as promising noncovalent, nonpeptidomimetic inhibitors with IC_50_ values between 15.06 and 51.81 μM. Furthermore, *in vitro* ADMET assays revealed these compounds to possess moderate aqueous solubility, low cytotoxicity, and high microsomal stability, making them excellent candidates for further development targeting SARS-CoV-2 PLpro.

Since the pandemic declaration by the World Health Organization in 2020, the SARS-CoV-2 virus, responsible for severe acute respiratory syndrome (COVID), has caused over 280 million cases and approximately 6 million deaths worldwide.^1^ Despite the development and administration of vaccines,^2^ the emergence of new variants resistant to these vaccines has raised concerns, which warrant the search for effective therapeutics against the virus.^3^

The current antiviral therapies focus on essential targets for the virus’s life cycle. Progress in the development of drugs targeting the main protease (Mpro) or 3C-like protease (3CLpro), such as paxlovid (nirmatrelvir/ritonavir),^4^ and the viral polymerase, such as remdesivir^5−8^ and molnupiravir,^5,6^ has offered a rapid treatment avenue. However, the challenges presented by viral mutations and drug resistance underscore the necessity for alternative strategies.^7^ In this regard, the papain-like protease (PLpro), which is conserved across various coronaviruses, plays a critical role in viral replication and immune evasion.^8,9^ Unlike highly mutable proteins such as the spike protein, PLpro’s conservation among SARS-CoV-2 variants positions it is a promising target for small-molecule inhibitors. Its essential function also diminishes the likelihood of resistance developing due to mutations that would compromise the enzyme’s activity.^10^ The catalytic site of PLpro cleaves a common motif, LXGG/X, present in nonstructural proteins NSP1/2, NSP2/3, and NSP3/4 proteins. This motif is critical for viral transcription and replication. Moreover, PLpro binding loop 2 (BL-2 Loop) controls access to the active site and is considered the binding site for the noncovalent inhibitor.^8,11^ The *N*-terminal ubiquitin-like domain of PLpro also acts as an antagonist to the innate immune pathway, playing a significant role in evading the host immune system.^9^ Numerous PLpro inhibitors have been documented in the literature,^11−15^ including the compound GRL0617^16^ (naphthalene scaffold) with a reported IC_50_ value of 2.1 μM, 2-phenylthiophene derivatives^17,18^ (IC_50_ ranging from 0.11 to 0.97 μM), and the RI173 compound^19^ (dimorpholine-thiuram disulfide scaffold) with an IC_50_ of 0.2 μM. More recently, SIMR30301, an octahydroindolo[2,3-*a*]quinolizine analogue,^20^ was discovered with an IC_50_ of 0.0399 μM. Another 85 biarylphenyl benzamide noncovalent PLpro inhibitors^21^ were identified that inhibited PLpro with *K*_i_ values from 13.2 to 88.2 nM. Among them, the lead compound Jun12682 inhibited the protease, deubiquitinase, and deISGylase activity of PLpro. *In vivo* experiments confirmed Jun12682’s activity against SARS-CoV-2, and its variants, and has proved it to be a promising oral SARS-CoV-2 antiviral candidate. Despite all efforts, there are still no approved drugs against SARS-CoV-2 targeting PLpro.

In this work, we have developed, validated, and applied computational approaches such as shape-based models, molecular docking, and similarity clustering as part of an integrated virtual screening campaign. Our primary goal was to strategically identify promising compounds from the Holistic Drug Discovery and Development Centre (H3D) in-house chemical library. The prioritized compounds represented diverse chemotypes, which were experimentally assessed for their potential as inhibitors of PLpro. From the most potent hit of the first round of compounds tested, an optimized naphthyridine series was synthesized and experimentally evaluated through enzymatic, differential scanning fluorimetry (DSF) and early absorption, distribution, metabolism, excretion, and toxicity (ADMET) assays. The general workflow is illustrated in Figure 1.

**Figure 1:**
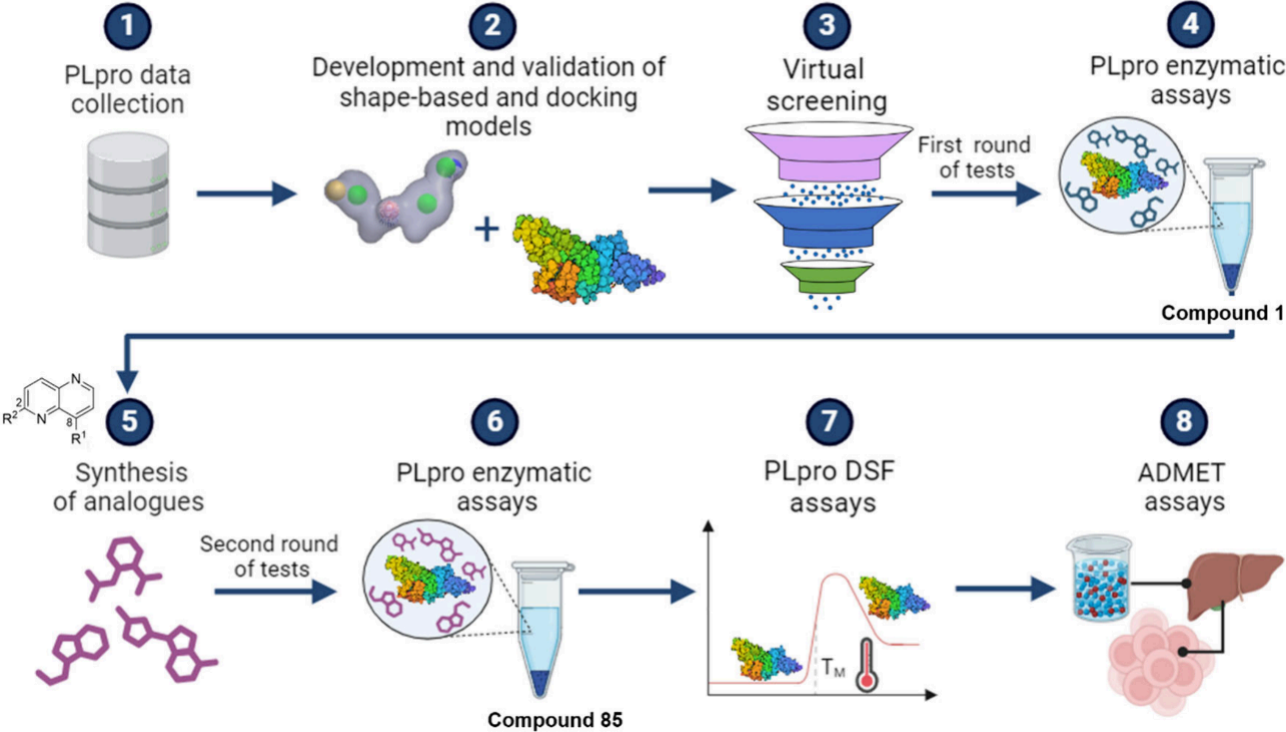
Integrated computational and experimental workflow applied for the discovery of novel noncovalent inhibitors of SARS-CoV-2 PLpro.

In order to perform the virtual screening (VS), the first step was to extensively collect data on PLpro inhibitors from the available literature to develop shape-based models (the first filter of the VS). This resulted in the identification of 63 PLpro active compounds. Shape-based models were then built to distinguish between active and inactive compounds against the SARS-CoV-2 PLpro. At the time of the literature survey, the most potent PLpro noncovalent inhibitor described was XR8-24, with an IC_50_ of 0.56 μM,^21^ and was selected as the core template for developing the shape-based models. The data set of XR8-24 derivatives was originally designed through a rational analysis (structure–activity relationship (SAR)) of a series of naphthalene scaffolds known for their activity against SARS-CoV-1 PLpro, despite their low metabolic stability in *in vivo* assays.^22−24^ As a result, the best model (Figure 2) presented the following key interactions with PLpro: two aromatic benzene rings (involved in π–π stacking interactions with the BL-2 loop Tyr268 residue), one hydrophobic thiophene (interacting with the hydrophobic “Groove” residues), one H-bond acceptor/donor amide group (interacting with the Asp164 and Gln269 residues), and one donor-cation (protonated azetidine) group (interacting with the Glu167 residue).

**Figure 2:**
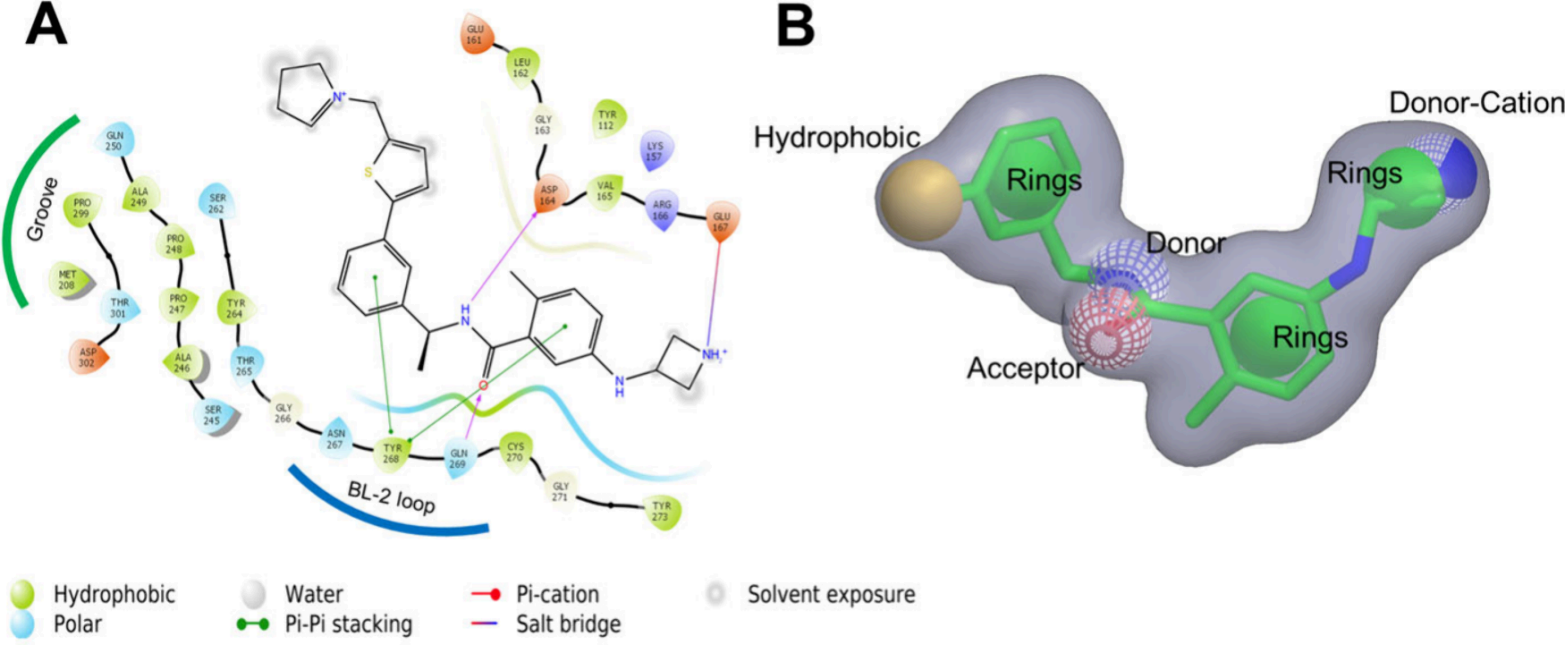
(A) 2D diagram of interactions of compound XR8-24 (BL2-Loop binding inhibitor) with PLpro residues of BL2-Loop. (B) Best shape-based model using the XR8-24 PLpro inhibitor as a template.

To validate the shape-based model, we gathered data on 14 inactive compounds against PLpro. We then simulated experimental conditions similar to those in a high-throughput screening campaign and generated 2254 decoys, which were added to the data set alongside the 14 inactive compounds from the literature.

The generated model achieved strong validation metrics, including an area under the ROC curve (AUC) of 0.95, an enrichment factor (EF) of 8.52, and a Boltzmann-enhanced discrimination of ROC (BEDROC) of 0.75, accurately identifying the top 10% of the tested data set from the literature (Table 1). Consequently, this shape-based model was employed as a filter during the virtual screening campaign.

**Table 1:** Statistical Metrics Were Obtained for the PLpro Shape-Based Model^a^

		TOP 1%		TOP 5%		TOP 10%	
query	AUC	EF	BEDROC	EF	BEDROC	EF	BEDROC
XR8-24-7	0.952	29.06	0.68	13.97	0.69	8.52	0.75

a AUC, area under the ROC curve; EF, enrichment factor; BEDROC, Boltzmann-enhanced discrimination of ROC.

Molecular docking calculations were performed with the Glide program^25−27^ to predict the ligands binding mode and to filter candidates during the virtual screening. The ligands were obtained from the H3D in-house library, and the 3D structure of SARS-CoV-2 PLpro was obtained from the Protein Data Bank^28^ (PDB ID 7LBS(18)). In order to assess the docking model reliability, we conducted the validation using the same data set collected and prepared from shape-based models, along with the crystal structure of SARS-CoV-2 PLpro PDB ID 7LBS.^18^ The results obtained from the top 10% of the list revealed satisfactory metrics, an AUC of 0.96, EF of 9.21, and BEDROC of 0.77, justifying the utilization of the protocol developed for docking purposes (Supporting Information Figure S1).

The H3D database (6892 compounds) underwent screening using the most effective SARS-CoV-2 PLpro shape-based model, resulting in the filtration of the top 10% of the list, comprising 689 compounds. Subsequently, docking calculations were performed, applying a threshold of GlideScore ≤ −6.95 kcal·mol^–1^ (filtering to 216 compounds). Then, a cluster analysis based on chemical similarity and a medicinal chemistry (MedChem)-based inspection allowed the selection of 81 compounds (Supporting Information File 2) with good scores. These 81 compounds presented important interactions with residues of the BL-2 Loop and warranted prioritization for experimental evaluation (Figure 3).

**Figure 3:**
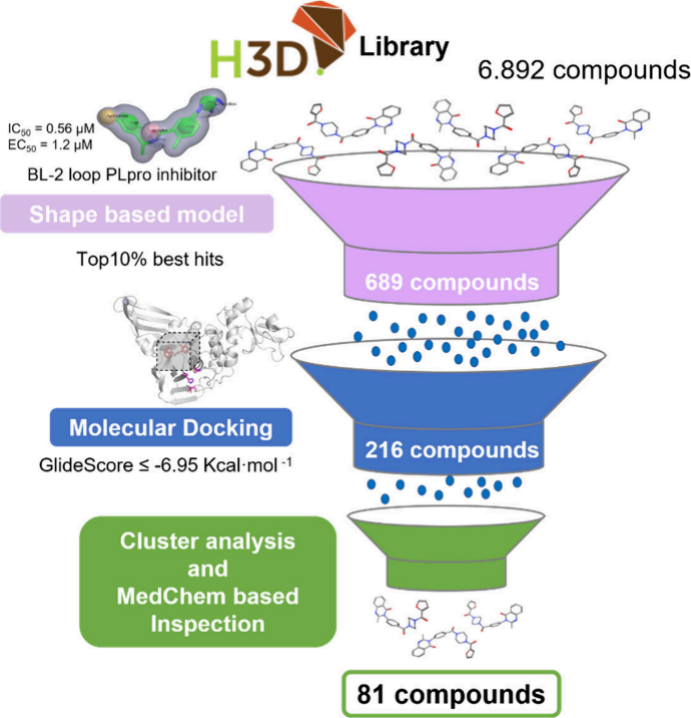
Schematic representation of the virtual screening workflow applied for the prioritization of potential candidates against SARS-CoV-2 PLpro.

The 81 compounds were evaluated *in vitro* for their inhibitory effects on PLpro, and nine of them exhibited some level of promising activity (Supporting Information Table S1). Among these, compound **1** showed an IC_50_ value of 73.60 ± 11.94 μM in the PLpro enzymatic assay (Figure 4A). In an inhibition test carried out with or without 0.01% Triton (Figure 4B), no significant differences were observed in the PLpro enzymatic activity, suggesting that this compound is not an aggregator. Kinetic characterization further suggested a competitive inhibition profile for **1**, with a *K*_i_ of 22.40 ± 6.7 μM, highlighting a moderate affinity for the target enzyme (Figure 4C). GRL0617 was used as a positive control for the enzymatic assays (Supporting Information Figure S2).

**Figure 4:**
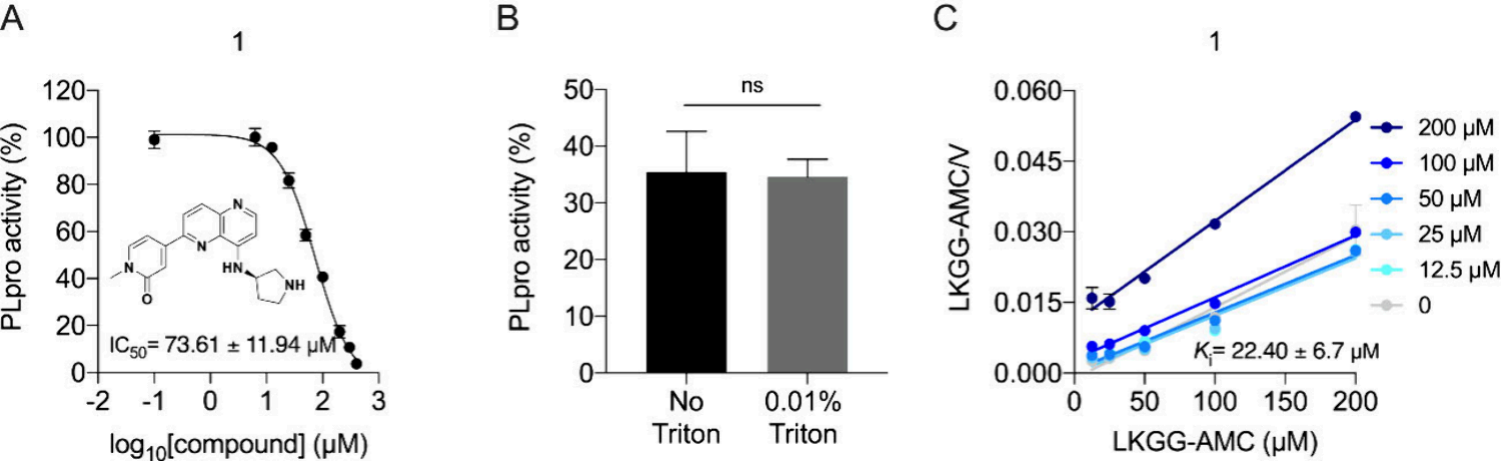
(A) Dose–response curve of **1** in the SARS-CoV-2 PLpro enzymatic activity. (B) Inhibition test of **1** in the absence and presence of 0.01% Triton. (C) Hanes–Woolf plot of compound **1**.

Considering the activity of **1**, a series of 30 naphthyridines were synthesized to explore the structure–activity relationships (SAR) between this naphthyridine chemical series and PLpro enzyme, with the goal of optimizing the inhibitor interactions with the PLpro enzyme and ultimately improving the SARS-CoV-2 antiviral activity (Supporting Information File 3). The 2,8-disubstituted-1,5-naphthyridines were synthesized using a linear synthetic route to allow diversification at the 2- and 8-positions of the naphthyridine core.^29,30^Scheme 1 (Supporting Information) shows the synthetic procedure utilized for the analogues of **1**.

All 30 synthesized naphthyridine analogues were screened in a second round of PLpro enzymatic assays. The four most potent compounds, **82**, **83**, **84**, and **85**, presented PLpro IC_50_ values ranging from 15.06 to 79.77 μM (Figure 5A–D), respectively. For the most potent compound **85**, we performed an inhibition test with and without 1 mM of 3-((3-cholamidopropyl) dimethylammonio)-1-propanesulfonate (CHAPS) detergent to verify whether the inhibition was due to aggregation. No significant difference was observed (Figure 5E). As observed for the initial hit **1**, compound **85** showed a competitive inhibition mechanism with a *K*_i_ of 22.93 ± 6.41 μM (Figure 5F).

**Figure 5:**
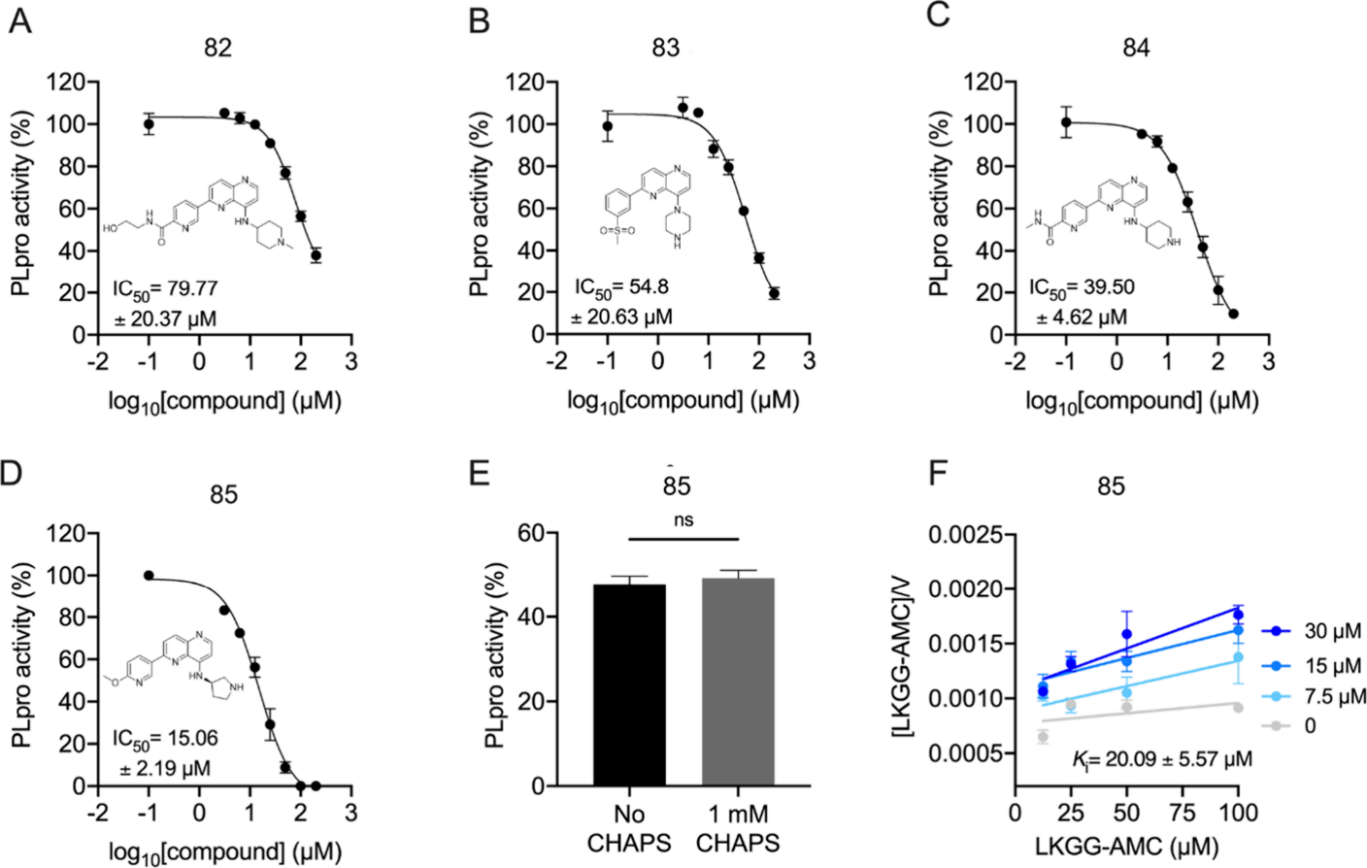
PLpro enzymatic assays for the selected compounds. (A, B, C, and D) Dose–response curves of compounds **82**, **83**, **84**, and **85**, respectively, in the SARS-CoV-2 PLpro enzymatic activity. (E) Compound **85** inhibition test in the absence and presence of 1 mM CHAPS detergent. (F) Hanes–Woolf plots of compound **85**.

The results of differential scanning fluorimetry (DSF) for the single-dose assays for **1** and its derivatives (Figure 6A–D) illustrate that changes in melting temperature are induced by the compounds. Notably, all compounds exhibited a thermoshift greater than 1 °C, indicating their binding to the PLpro protein. To further validate the specificity of these interactions, dose–response assays were conducted. The graphical representations of these assays show that compound **1** and its derivatives produce a clear dose–response curve, confirming specific interactions with the PLpro protein when compared to the positive control, GLR0617 (Figure 6B). Additionally, upon comparison of the outcomes derived from both enzymatic and DSF assays, an observable correlation emerges between the potency of compounds and their corresponding thermoshift (Figure 6C). This correlation can be leveraged to optimize the compound screening process.

**Figure 6:**
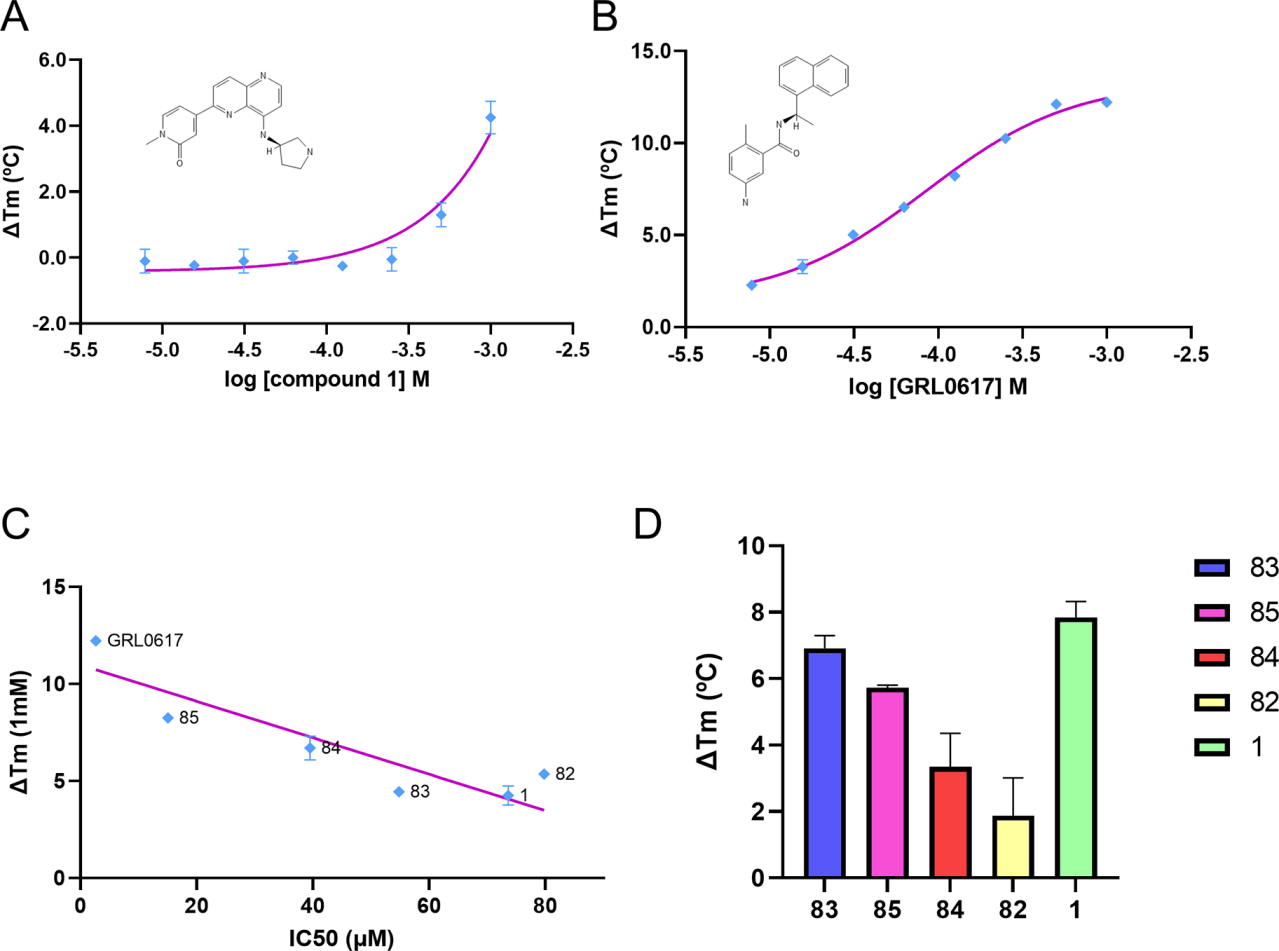
Differential scanning fluorimetry (DSF) analysis displaying the dose–response curve for compound **1** (A) compared to the noncovalent inhibitor GRL-0617 (B), which serves as a positive control. The *x* axis indicates the compound concentration (log *M*), while the *y* axis illustrates the thermoshift (°C). Panel C shows the correlation between potency, as measured by IC_50_ values (μM), and the corresponding thermoshift (°C) from biochemical assays. Last, panel D presents a bar graph depicting the single-dose thermoshift (Δ*T*_m_) for compounds **1**, **82**, **83**, **84**, and **85**. Error bars represent the standard deviation of the measurements, providing insight into the reproducibility and reliability of the data.

The results of the *in vitro* aqueous solubility, cytotoxicity, and microsomal stability properties of the five most promising naphthyridine derivatives (compounds **1**, **82**, **83**, **84**, and **85**) are presented in Table 2. All compounds exhibited high aqueous solubility (175–200 μM), which is beneficial for potential oral administration and crucial for bioavailability.

**Table 2:**
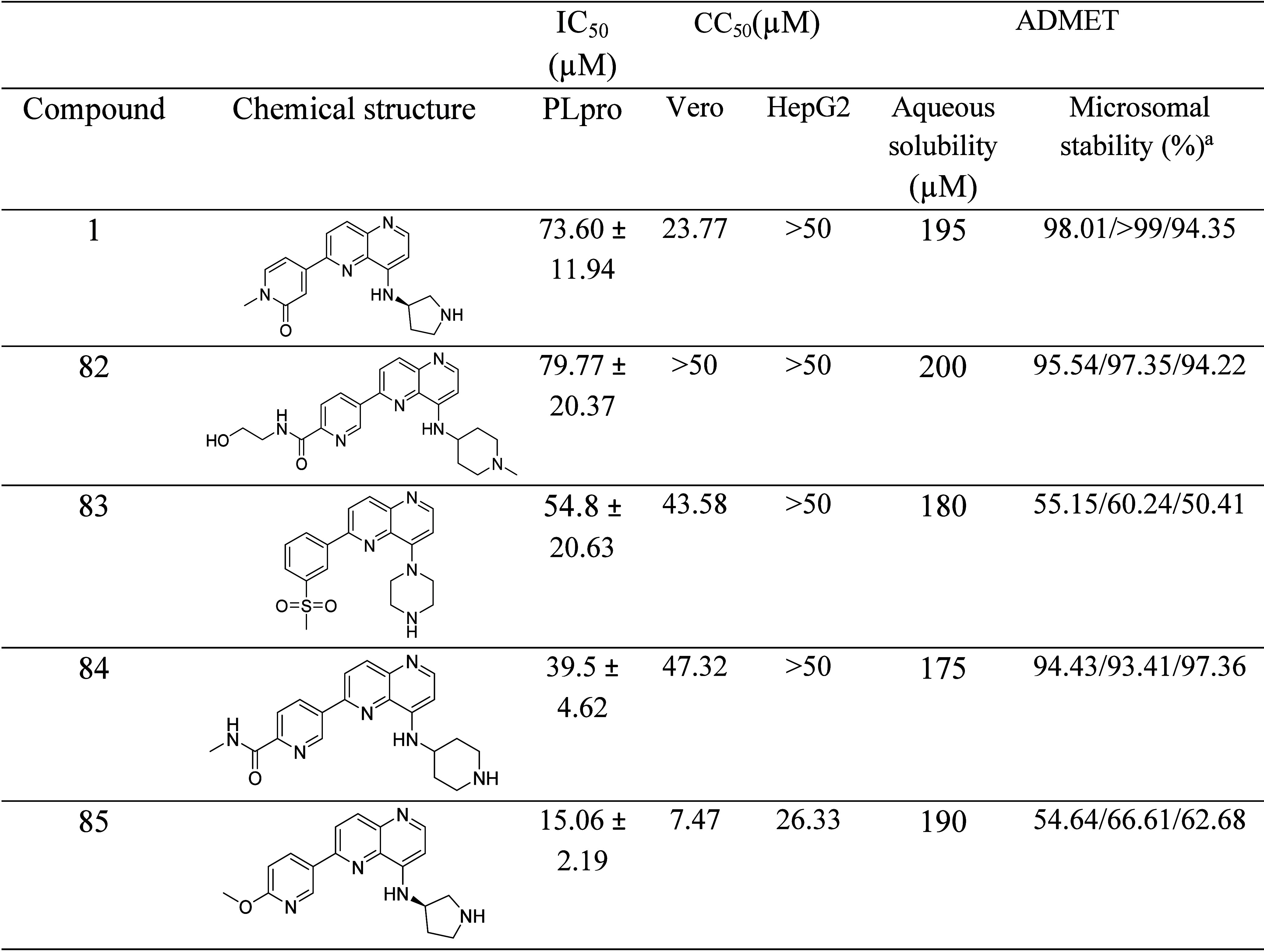
*In Vitro* Enzymatic and ADMET Results for Selected Naphthyridine Analogues

a % remaining after 30 min, human/mouse/rat. Data from Kandepedu and collaborators.^29^

All five naphthyridine compounds were evaluated for cytotoxicity against two mammalian cell lines, namely, Vero and HepG2, respectively (Table 2). The two most potent PLpro inhibitors (compounds **1** and **85**) demonstrated 2–3-fold higher toxicity against the Vero cell line (CC_50_ = 23.77 and 7.47 μM, respectively) and compounds **83** and **84** were equipotent against Vero cells. On the other side, all compounds demonstrated weaker toxicity against HepG2, indicating a more favorable safety profile against the human cell line. The difference in cytotoxicity observed between HepG2 and Vero cells can be attributed to the distinct biological characteristics of these two cell lines. HepG2 cells are derived from human liver carcinoma and possess more efficient detoxification mechanisms, including cytochrome P450 enzymes, which may metabolize or neutralize toxic compounds. In contrast, Vero cells are derived from monkey kidney epithelial cells and lack many of the metabolic pathways found in liver cells.^31−33^ As a result, compounds that are nontoxic to HepG2 cells may accumulate to toxic levels in Vero cells, leading to cytotoxic effects. This disparity underscores the importance of using different cell lines to assess cytotoxicity, as it helps capture a broader range of potential toxic responses in varying cellular environments.^32^ Future SAR studies should include efforts to understand and diverge the PLpro activity and cytotoxicity relationship.

Microsomal stability was tested in human, mouse, and rat liver microsomes over a 30 min period (Table 2). Compounds retaining over 50% of their initial concentration after 30 min are considered to have good metabolic stability, with ideal stability being over 85%. Compounds **1**, **82**, and **84** demonstrated high metabolic stability across the mouse, rat, and human liver microsomes, with more than 90% of the compound remaining after 30 min. In contrast, compounds **83** and **85** showed lower stability, particularly against human microsomes, with only about 50% remaining.

When the data in Table 2 for compounds **1** and **85** are reviewed, the replacement of the 1-methylpyridin-2(1H)-one with 2-methoxypyridine led to a 5-fold improvement in PLpro activity but at a cost to metabolic stability in human, rat, and mouse liver microsomes. This may be due to an increased susceptibility to demethylation of the methoxy substituent in the presence of microsomes. In comparison, compound **83**’s apparent loss in metabolic stability in microsomes appears to be attributed to the piperazinyl group. To better understand the metabolic fate of compounds **83** and **85**, liquid chromatography-tandem mass spectrometry (LC-MS/MS) metabolic identification (MetID) could be done in future work to identify the metabolites. Further SAR expansion at these positions would also provide valuable information to understand the metabolic fate for the series.

In this study, an initial SAR exploration was carried out for this series by keeping the naphthyridine core constant and exploring diverse substituents at the 8′- (R_1_) and 2′- (R_2_) positions on the naphthyridine core (Figure 7).

**Figure 7:**
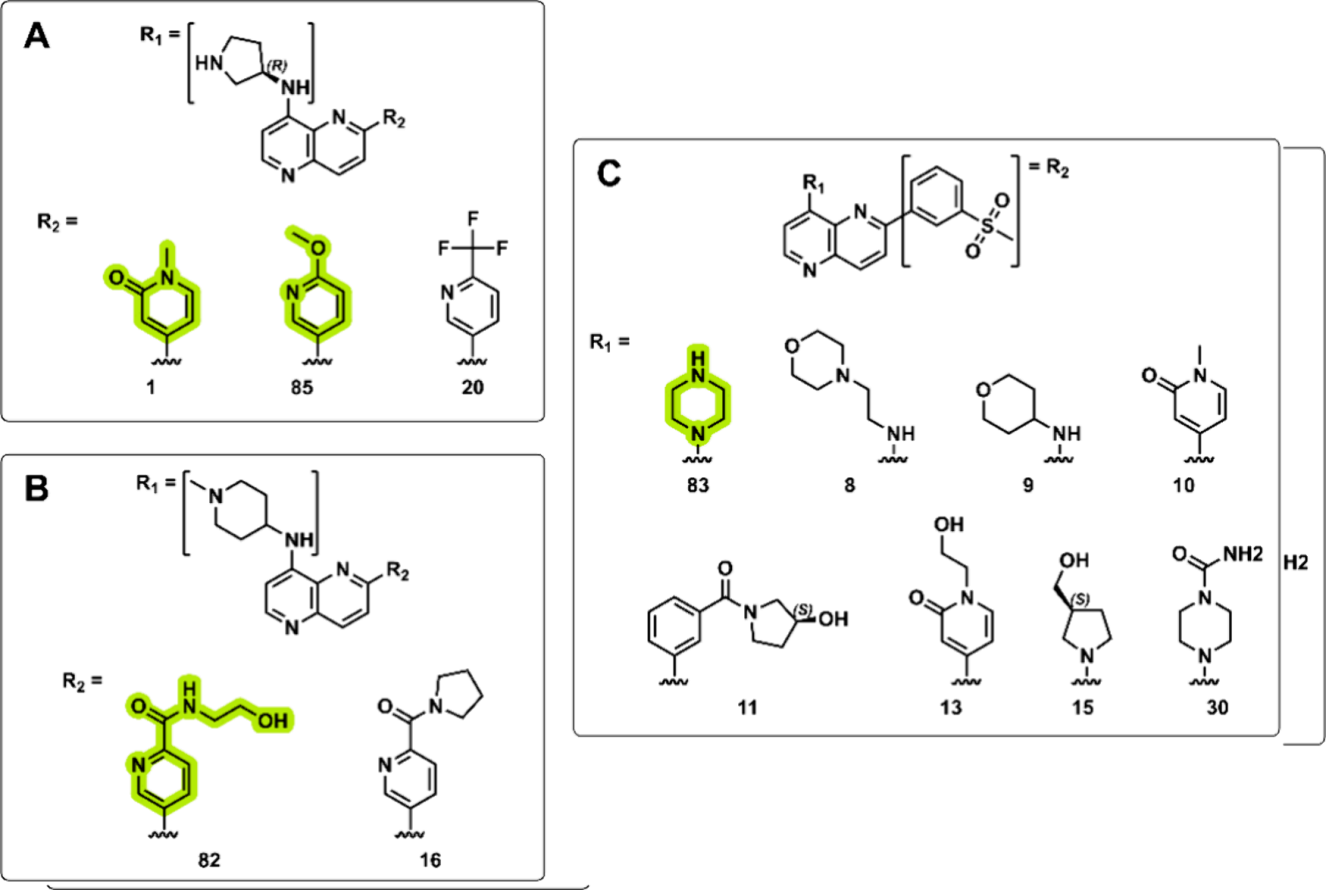
SAR analysis of naphthyridine derivatives. (A) R_1_ = (3*R*)-3-aminopyrrolidin-1-yl group with exploration at R_2_. (B) R_1_ = (1-methylpiperidin-4-yl)aminyl group with exploration at R_2_. (C) R_2_ = 4-methanesulfonylphenyl with exploration at R_1_. Substituents that rendered favorable improvements in PLpro IC_50_ are highlighted in green.

When R_1_ is a 3-aminopyrrolidinyl group, the substitution of R_2_ with a pyridone (**1**) or methoxypyridine group (**85**; Figure 7A) resulted in IC_50_ values of 73.60 and 15.06 μM, respectively. This 5-fold improvement in PLpro activity may be the result of a more optimal orientation of the pyridinyl-*N* (moving from the 4′ to 3′ position) and the increase of the electron donating character of the *N*, which enhances the proposed interaction with the Arg166 residue (Figure 8). This can be further validated by comparison with **20** where the highly electron-withdrawing *ortho*-trifluoromethyl group would significantly reduce the electron-donating character of the pyridinyl-*N*. This would compromise the key electron-donating interaction with Arg166, which could potentially predict and agree with the observed complete loss in PLpro activity. The pyrrolidine ring at R_1_ of compound **85** interacts with Glu167 through both a hydrogen bond and a salt bridge (Figure 8), as predicted by docking calculations. At R_2_, a 6-methoxypyridine interacts by accepting a hydrogen from Arg166, and the ring engages in π-stacking interactions with Tyr264. In contrast, the 1-methyl-2-oxo-1,2-dihydropyridin-4-yl substituent (compound **1**) is more polar and exhibits reduced aromaticity due to the partial conjugation of the ring caused by the oxo group. This disruption seems to weaken its interaction with the enzyme’s active site, leading to a higher IC_50_ (73.60 μM). Compound **85** also presents interaction with Glu167 (Figure 8). The flexibility in R_2_ maintains hydrophobic interactions with the BL-2 loop. On the other hand, substituents such as 6-(trifluoromethyl)pyridin-3-yl (**20**; Figure 7A), despite its high lipophilicity and electronegative character, showed no activity.

**Figure 8:**
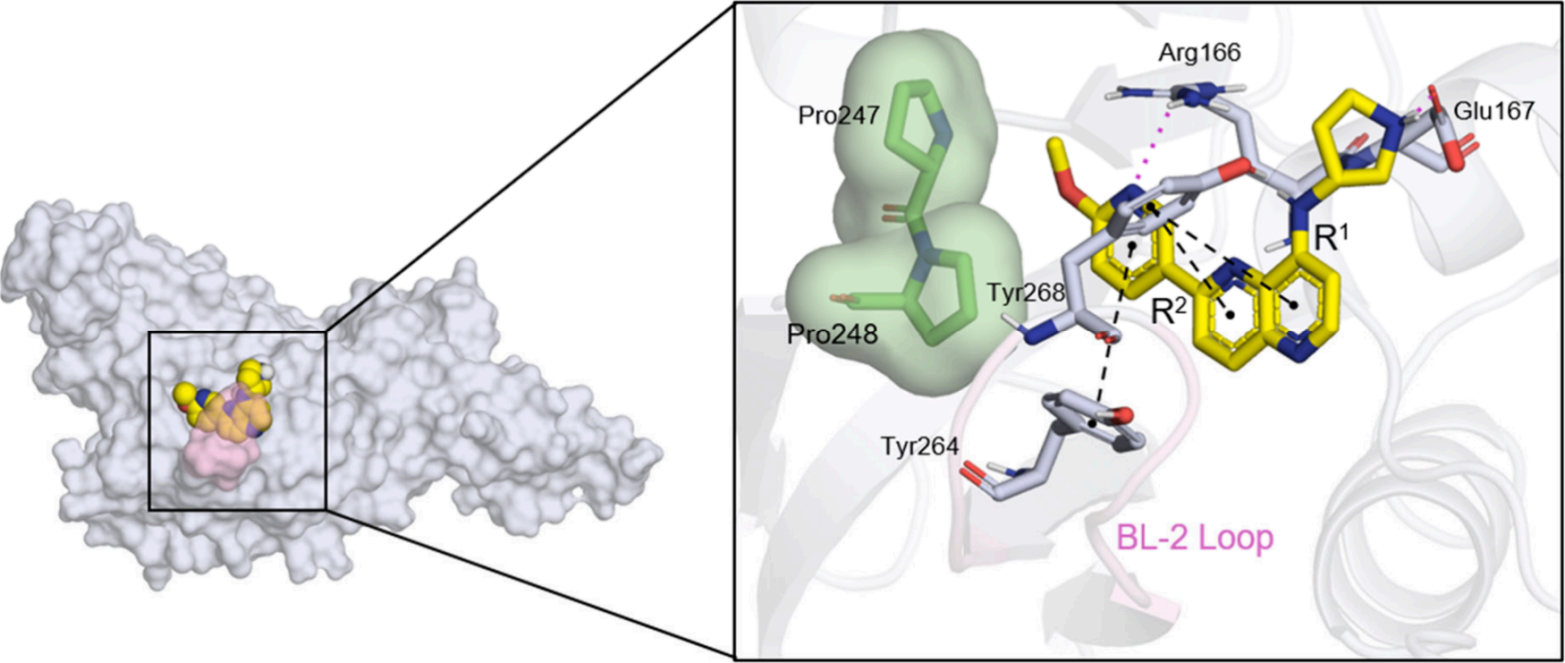
Docking pose of compound **85**, the most promising inhibitor of SARS-CoV-2 PLpro identified in this study, along with its ligand–protein interactions. On the left, the papain-like protease (PLpro) is illustrated using a blue-white surface representation, with the BL-2 loop highlighted in light pink and the ligand shown as yellow spheres. The right panel provides a zoomed-in view of the ligand–protein interactions, featuring a green surface to indicate hydrophobic interactions, pink dashed lines representing hydrogen bonds, and black dashed lines denoting π–π stacking interactions. This detailed visualization underscores the binding characteristics of compound **85** and its potential as an effective therapeutic agent.

Where R_1_ is a (1-methylpiperidin-4-yl)aminyl group, substituting R_2_ with a 6-[(2-hydroxyethyl)carbamoyl]pyridin-3-yl (**82**; Figure 7B) resulted in moderate activity (IC_50_ of 79.77 μM) against the PLpro enzyme. This substituent is more polar and sterically bulky and may impact its ability to make the key hydrophobic interactions with the enzyme (Supporting Information Figure S3). Similarly to **85**, the pyridinyl-*N* is in the proposed more favored 3′-position, which could lead to the weak retention of PLpro activity. In contrast, the substitution of R_2_ with 6-[(2-hydroxyethyl)carbamoyl]pyridin-3-yl (**16**; Figure 7B) at the same position resulted in no measurable activity. This moiety is more lipophilic and rigid, which may hinder binding through polar interactions.

While retaining R_2_ as a 4-methanesulfonylphenyl group, the substitution of R_1_ with a piperazin-1-yl (**83**; Figure 7C) was favorable, yielding an IC_50_ of 54.81 μM. Similar to compounds **1**, **82**, and **85**, a basic group at position R_1_ that is able to make the key salt bridge interactions with the PLpro Glu167 residue (Supporting Information Figure S3) is critical for activity. In contrast, nonbasic substituents that are not able to make the critical salt-bridge interaction with Glu167 at the same position, such as (oxan-4-yl)aminyl (**9**), (1-methyl-2-oxo-1,2-dihydropyridin-4-yl)methyl (**10**), 3-[(3S)-3-hydroxycyclo-pentanecarbonyl]phenyl (**11**), [1-(2-hydroxyethyl)-2-oxo-1,2-dihydropyridin-4-yl]-methyl (**13**), (3R)-3-(hydroxymethyl)pyrrolidin-1-yl (**15**), and 4-carbamoyl-piperazin-1-yl (**30**; Figure 7C), did not exhibit any activity. For **8**, the increased steric bulk and altered electronic environment due to the oxygen in the morpholine ring may contribute to the lack of activity. These findings provide valuable insights into the structural elements that either contribute to or hinder the inhibitory effects of the compound on PLpro.

In conclusion, our integrated approach that combined computational strategies and experimental validation unveiled a novel class of noncovalent naphthyridine inhibitors of the SARS-CoV-2 PLpro enzyme. From 81 prioritized virtual hits of the H3D database, we identified the naphthyridine compound **1** that exhibited moderate activity against PLpro. The limited structural optimization of this compound led to the identification of compound **85** with increased potency against PLpro. Moreover, the naphthyridine series exhibited promising ADMET profiles with high aqueous solubility and high to moderate microsomal stability. Cytotoxicity was identified as a possible concern to developability of the naphthyridine series as a treatment for SARS-CoV-2. Future work should include SAR exploration to understand the observed unfavorable enzymatic vs cytotoxicity relationship and identify SAR trends that could diverge the series enzymatic activity from the cytotoxicity. Overall, these findings provide a foundation for the continued exploration of the naphthyridine scaffold as a potential antiviral therapeutic agent in future hit-to-lead optimization efforts. The discovery of noncovalent inhibitors of SARS-CoV-2 PLpro provides new mechanistic insights into the inhibition of this critical viral enzyme. Understanding these interactions at the molecular level can guide the design of more potent and selective inhibitors. Moreover, given the conserved nature of PLpro across different coronaviruses, the inhibitors identified in this study could potentially be effective against a broad spectrum of coronaviruses including those that may emerge in the future.

## Data Availability

The data curation script utilized in this study is available at https://github.com/LabMolUFG/cheminformatics_pipeline.

## Supporting Information Available

Detailed computational procedures, chemical structures of the 30 naphthyridines, experimental procedures, supplementary figures, and compounds’ characterizations (PDF)
Data for compunds 1–81 (XLSX)
Chemical structures (XLSX)

## Abbreviations

3CLpro: 3C-like protease
ACE2: angiotensin-converting enzyme 2
ADMET: absorption, distribution, metabolism, excretion, and toxicity
AUC: area under the curve
BEDROC: Boltzmann-enhanced discrimination of receiver operating characteristic
BL-2: binding loop 2
CHAPS: 3-((3-cholamidopropyl) dimethylammonio)-1-propanesulfonate
COVID-19: coronavirus disease 2019
DMSO: dimethyl sulfoxide
DSF: differential scanning fluorimetry
EC_50_: 50% effective concentration
EF: enrichment factor
IC_50_: half maximal inhibitory concentration
*K*_i_: inhibition constant
LC-MS/MS: liquid chromatography-tandem mass spectrometry
MedChem: medicinal chemistry
MetID: metabolic identification
Mpro: main protease
NSP: nonstructural proteins
PDB: Protein Data Bank
PLpro: papain-like protease
SARS-CoV-2: severe acute respiratory syndrome coronavirus 2
VS: virtual screening
WHO: World Health Organization
