# Supplementary material for: Discovery of Non-Covalent Inhibitors for SARS-CoV-2 PLpro: Integrating Virtual Screening, Synthesis, and Experimental Validation
Source: ACS Med Chem Lett. 2024 Dec 2;15(12):2140–9. doi: 10.1021/acsmedchemlett.4c00420 (PMC11647681; doi:10.1021/acsmedchemlett.4c00420)
Supplement: Supplementary file 1 — ml4c00420_si_001.pdf [file ml4c00420_si_001.pdf]

Supplementary Information

Unveiling Novel Noncovalent Inhibitors Against SARS-CoV-2 PLpro: An Integrated Approach of Virtual Screening, Synthesis, and Experimental Validation

Bruna K P Sousa[1,2], Melina Mottin[1,2,4], Donald Seanego[3], Christopher D Jurisch[3], Beatriz S A Rodrigues[4], Verônica L S da Silva[4], Milene Aparecida Andrade[4], Gilberto S Morais Junior[4], Diogo F Boerin[1,5], Thamires Q Froes[1,5], Flávia Nader Motta[4,6], M. Cristina Nonato[1,5], Izabela D M Bastos[4], Kelly Chibale[3,8,9], Richard K Gessner[3] and Carolina Horta Andrade[1,2,7*]

1. Center for the Research and Advancement in Fragments and Molecular Targets (CRAFT), Faculdade de Ciências Farmaceuticas de Ribeirão Preto, Universidade de São Paulo, Ribeirão Preto, SP, Brazil.
2. Laboratory for Molecular Modeling and Drug Design (LabMol), Faculdade de Farmácia, Universidade Federal de Goiás, Goiânia, GO, Brazil.
3. Holistic Drug Discovery and Development Centre (H3D), University of Cape Town, South Africa.
4. Pathogen-Host Interface Laboratory, Department of Cell Biology, University of Brasilia, Brasilia, Brazil.
5. Laboratório de Cristalografia de Proteínas, Faculdade de Ciências Farmacêuticas de Ribeirão Preto, Universidade de São Paulo, Ribeirão Preto, SP, Brazil.
6. Faculdade de Ceilândia, Universidade de Brasília, Brasília, DF, Brazil.
7. Center for Excellence in Artificial Intelligence (CEIA), Instituto de Informática, Universidade Federal de Goiás, Goiânia, GO, Brazil.
8. South African Medical Research Council Drug Discovery and Development Research Unit, University of Cape Town, South Africa.
9. Institute of Infectious Disease and Molecular Medicine, University of Cape Town, South Africa.

Table of Contents

Computational procedures 3
Experimental procedures 6
Supplementary Tables 10
Supplementary Figures 12
Supplementary Results 14
Compounds characterization 16
References 41

Computational procedures

Dataset collection, curation and preparation

Compounds were collected from the literature compounds that were tested against the SARS-CoV-2 PLpro, reported by Shen and collaborators [1]. The initial data was compiled and integrated, and curated according to the protocol by Fourches and collaborators [2,3]. Briefly the hydrogens were explicitly included, while counter ions, inorganic salts, polymers, mixtures, and organometallic compounds were excluded. Additionally, specific chemotypes such as aromatic and nitro groups were standardized. A threshold of activity of the inhibitors was set as $IC_{50} \leq 10$ μM as active (63 compounds), and $IC_{50} \geq 10$ μM as inactive compounds (14 compounds). Duplicates analysis was performed following the criteria: (*a*) entries with identical reported outcomes were consolidated, keeping only one, while the redundant entry was eliminated, and (*b*) if duplicates exhibited inconsistent biological activity, both entries were excluded from the dataset. The final dataset contained 77 compounds.

In order to increase the chemical space of the collected dataset, decoys were generated in proportion for each inhibitor 36 decoys. The dataset of actives, inactives and decoys had the appropriate hydrogen protonation state on neutral pH (7.4) setup at Open Babel program [4]. Next were generated for each molecule 200 conformers at OMEGA [5] program, and estimated the AM1BCC charges [6,7] using QUACPAC software [8]. Then we build and validate shape-based models using the ligand conformation, extracted from the PDB (ID: 7LBS [1]) and used as a query at ROCS v. 3.4.2.1 program [9,10].

Shape based model development and validation

To distinguish actives from inactives, a shape-based model was constructed and validated. To assess the predictive performance of the queries the following metrics were analyzed: Receiver Operating Characteristic (ROC) curve, that provides a graphical description of the true positive rate (sensitivity) minus false positive rate (1-specitivity); the Area Under the ROC Curve (AUC), is the probability of an inhibitor to be ranked first than an inactive when compared to a random selection by the query [10,11]; the Boltzmann-Enhanced Discrimination of ROC (BEDROC), which uses exponential decay function to attach weights to actives that ranked in the first positions of the list [12]; and Enrichment Factor (EF), that evaluates the fraction of actives found at the Top n% of the ranking

compared with a random selection [8,10]. The statistics metrics were calculated by the following equations:

$$AUC = \sum i[(Se_{i+1})(Sp_{i+1} - Sp_i)] \quad (1)$$

$$BEDROC = RIE \frac{R_\alpha \sin\left(\frac{\alpha}{2}\right)}{\cosh\left(\frac{\alpha}{2}\right) - \cosh\left(\frac{\alpha}{2 - \alpha R_\alpha}\right)} + \frac{1}{1 - e^{\alpha(1-R_\alpha)}} \approx \frac{RIE}{\alpha} + \frac{1}{1 - e^{\alpha'}} \quad (2)$$

$$if\ \alpha R_\alpha \ll 1\ and\ \alpha \neq 0$$

$$EF^{x\%} = \frac{\frac{Hits^{x\%}selected}{N^{x\%}selected}}{\frac{Hits_{total}}{N_{total}}} \quad (3)$$

Protein preparation for docking calculations

The 3D protein structure of SARS-CoV-2 PLpro (PDB ID: 7LBS [1]) was processed in the Protein Preparation Wizard module [13,14] in Maestro [15]. The protocol includes addition of hydrogen atoms, calculation of ionization states at physiological pH (7.4 ± 0.5) using Epik program [16,17] removal of structural waters with a distance greater than 3 Å from the side chains. The protein hydrogen bonds assignments and protonation states were refined using PROPKA [17], and finally a restrained minimization was performed using the OPLS-2005 force field [18,19].

Ligand preparation for docking calculations

The preparation of the ligands (either H3D library and SARS-CoV-2 PLpro dataset) was carried out in the LigPrep module in Maestro [20] which the ionization states were set at physiological pH (7.4 ± 0.5) using Epik program [16], conformers were generated, the stereoisomerism of the molecule was retained, and the geometry was minimized using the OPLS-2005 force field.

Grid generation for docking calculations

The 3D coordinates of the grid were constructed using the Receptor Grid Generation module [21] at the region reported in the literature at the BL-2 Loop [22] using the coordinates X: 8.58 Å, Y: -7.99 Å, Z: 31.97 Å, and have a size of 10 Å.

Docking calculations and validation protocol

The docking calculations were carried out at Glide program [21,23,24], considering the ligand flexible and the protein rigid, executed in standard precision (SP) whose score function uses an exhaustive sampling search, recommended for virtual screenings campaigns [24]. For the VS we screened the in-house H3D library.

For the docking validation protocol, we utilized with the same SARS-CoV-2 PLpro active and inactive dataset used in shape-based models prepared and the 3D protein structure of PLpro, executed in standard precision (SP). The statistical metrics AUC, EF and BEDROC were computed in the KNIME [25,26] in-house workflow [27], in order to assess the robustness of the docking program.

Virtual screening

After validating the shape-based and molecular docking protocol, the virtual screening was carried out using the H3D library, which totaled more than six thousand compounds. The data were compiled and processed following the mentioned protocol for ligand preparation. The first filter was the best shape-based model for PLpro, where the Top 10% of the list went to the molecular docking calculations.

For the final selection, cluster analysis was performed using DataWarrior program [28]. The compounds were clusterized based on chemical structure similarity (> 80%) using SkelSpheres chemical descriptor. A final a medicinal chemistry MedChem-based inspection of the docking pose was performed to prioritize candidates to be purchased and validated on *in vitro* assays.

Structure activity relationships analysis of naphthyridines

A SAR study of all tested compounds was developed based on the substituents R^1 and R^2 added to the naphthyridine scaffold and their interaction with BL2-Loop of PLpro, predicted by molecular docking. The analysis of the protein-ligand interactions was performed using Maestro 2D diagram interaction and PLIP webserver [29]. Based on the accounting and analysis of interaction types, we correlated these findings with the experimentally obtained IC_{50} values.

Experimental procedures

Chemical synthesis

The synthesis of the naphthyridine derivatives was performed following the procedures described by Kandepedu and collaborators (2018) [30] and Dziwornu and collaborators (2024) [31]. The Sup. Info. **Scheme 1** shows the synthetic procedure utilized for the analogues of **compound 1**.

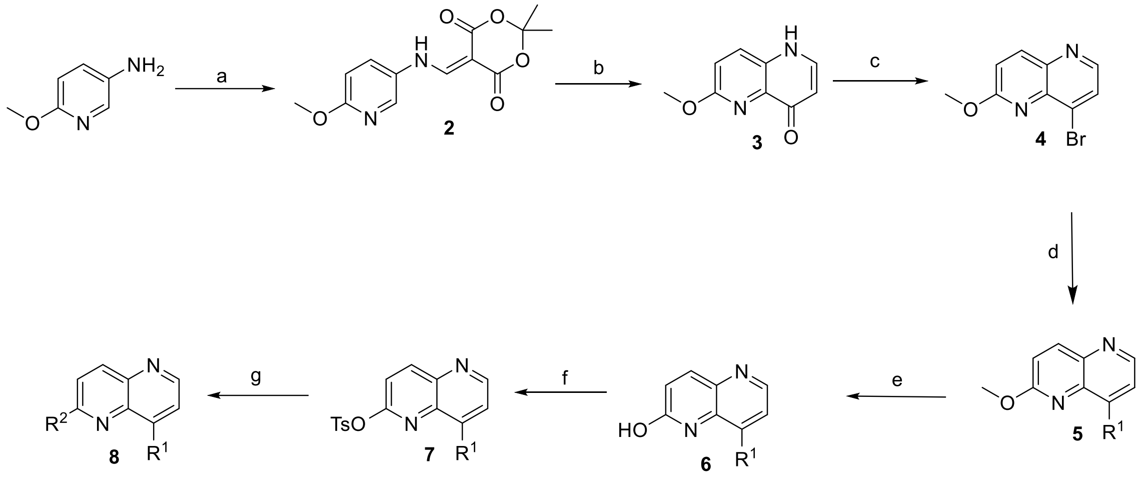

Scheme 1: Reagents and conditions: (a) 2,2-dimethyl-1,3-dioxane-4,6-dione, trimethoxymethane, ethanol, 105 °C, 12 h, 80%; (b) Dowtherm A, 220 °C, 64%; (c) PBr_3, DMF, 0 °C to rt, 87%; (d) R^1 -$B(OH)_2$, $PdCl_2$(dppf), Cs_2CO_3, dioxane, 95 °C, 41–91%; or R^1 -appropriate amine, Cs_2CO_3, DMF, 110 °C, 12 h, 45–71%; (e) HBr, 85 °C, 89–95%; (f) $POBr_3$, 60 to 120 °C, 55–91% or p-toluenesulfonyl chloride, triethylamine, 4-dimethylaminopyridine (DMAP), dichloromethane (DCM), 20–30 °C, 2 h, 78%; (g) R^2 -$B(OH)_2$, $PdCl_2$(dppf), Cs_2CO_3, dioxane, 95 °C, 8–64%; or R^2 -$B(OH)_2$, $Pd_2(dba)_3$, PCy_3, K_3PO_4, dioxane, 125 °C, 17–23%; or R^2 -appropriate amine, Cs_2CO_3, DMF, 110 °C, 12 h, 30–41%; or R^2 -appropriate aromatic amine, $Pd_2(dba)_3$, BrettPhos, Cs_2CO_3, toluene, tert-butanol, 115 °C, 34%.

Shortly, starting from a commercially available 6-methoxypyridin-3-amine, which undergoes condensation with Meldrum's acid and trimethoxymethane to afford intermediate **2**, followed by subsequent thermal cyclisation to form **3**. Bromination of **3** using phosphorus tribromide (PBr_3) at 0 °C resulted in key intermediate 8-bromo-2-methoxy-1,5-naphthyridine **4**, which enabled diversification at 8-position of the 1,5-

naphthyridine ring. This key intermediate was then subjected to palladium-catalyzed Suzuki cross-coupling reaction or nucleophilic aromatic substitution using commercially available boronic acids or amines, respectively, to give intermediate **5**. Intermediate **6** was achieved by *O*-demethylation using HBr followed by second bromination using $POBr_3$ or tosylation to give **7**. This intermediate **7** was either subjected to a second Suzuki cross-coupling or S_NAr substitution reaction using boronic acids or amines, respectively, to furnish target compounds **8**.

All compounds tested for enzymatic assays were confirmed to have ≥95% purity. Liquid chromatography (LC) purity traces were performed using methods reported in Supplementary Material. The detailed spectroscopic and purity data (LC-MS, ^1H and ^{13}C NMR) are provided in the Supplementary Material.

SARS-CoV-2 PLpro expression and purification

The recombinant SARS-CoV-2 PLpro was produced as previously described with some modifications [32]. Briefly, SARS-CoV-2 PLpro gene was cloned into the pET-19b expression vector. The plasmid was transformed into *Escherichia coli* strain BL21(DE3). The expression of SARS-CoV-2 PLpro recombinant protein was induced by addition of 0.1 mM IPTG and 1.0 mM $ZnCl_2$ for 18h at 18 °C. To purify the recombinant enzyme, cells were harvested, lysed with BugBuster® (Merck) and centrifuged at 16,000 *g* for 20 min at 4 °C. The supernatant was submitted to affinity chromatography on a nickel-agarose resin (Sigma). After the resin had been extensively washed with 50 mM Tris-HCl (pH 8.0), 0.5 M NaCl and 5 mM imidazole, bound SARS-CoV-2 PLpro was eluted with the same buffer containing 80 mM imidazole. The purified enzyme was dialyzed against 20 mM Tris-HCl pH 7.3, concentrated and stored at -80 °C.

SARS-CoV-2 PLpro inhibition assay

The IC_{50} values were determined from 400 to 3.125 μM of inhibitor plus the positive control. Inhibition assays were performed, in triplicate, with 30 μM SARS-CoV-2 PLpro recombinant protein in 20 mM Tris-HCl pH 7.3, 1 mM EDTA, during 15 min. The substrate LKGG-AMC was added to a 100 μL reaction mixture at a final concentration of 20 μM. Subsequently, AMC released fluorescence was measured under excitation at 355 nm and emission at 460 nm in kinetic mode for 20 min using the SpectraMax® M5 Microplate Reader. The curves were fitted in a four-parameter logistic (4 PL) non-linear

regression model using GraphPad Prism® version 8 software (GraphPad Software, www.graphpad.com) for the calculation of IC_{50}. Mechanism of inhibition and K_i values were calculated from at least four different inhibitor concentrations, chosen according to previously calculated IC_{50} for each compound, and at least four substrate concentrations (100 - 6.25 µM). For the compounds selected to follow with inhibition mode characterization, an inhibition test with and without 1 mM CHAPS or 0.01% Triton was performed to confirm that they are not PAINS. All kinetic parameters were determined from the collected data by nonlinear regression employing the GraphPad Prism® enzyme kinetics module and Hanes-Woolf plots [33].

SARS-CoV-2 PLpro differential scanning fluorimetry assays

The expression and purification of the PLpro was performed as described by Freire and co-workers [34]. DSF assays were performed on Agilent Mx3005P QPCR System (Agilent Technologies, Santa Clara, USA) with excitation and emission wavelengths of 492 nm and 610 nm, respectively. All assays were carried out in triplicate on a 96-well PCR plate (PCR plates 96 well BioRadVR), manually sealed with transparent capping strips (Flatcap strips BioRadVR). The plates were centrifuged for 2 min, 2000 rpm, at 25ºC and then the fluorescence of SYPRO® orange (S6650) was monitored while the plate was heated from 25 to 85ºC, in increments of 1ºC per minute.

Prior to compound screening, protein concentration and DMSO concentration were screened to optimize PLpro stability throughout the assay. Briefly, signal-to-noise ratio was calculated with protein concentration ranging from 1 to 20 µM. Next, the influence of DMSO (2.5, 5, 10% v/v) on the Tm values was investigated. The screening of the naphthyridines derivatives was carried out under optimized conditions, at a single concentration (1000 µM final concentration). For dose-response assay, the concentration of the compounds varied from 1 mM to 0,016 mM. An equivalent volume of DMSO was used as negative-inhibition control. All experiments were carried out in triplicate.

Raw fluorescence data were recorded within the Agilent Mx3005P QPCR Software, and then exported to NAMI [35] for Tm calculation by the first-derivative method. The graphs were plotted using GraphPad Prism® version 9.3.0 software (GraphPad Software, www.graphpad.com).

Cytotoxic, microsomal stability and aqueous solubility assays

The cytotoxicity, microsomal stability, and aqueous solubility assays were performed as previously described by Kandepedu and collaborators (2018) [30] and Dziwornu and collaborators (2024) [31].

Supplementary Tables

Supplementary Table S1. Inhibitory activity (IC_{50}) of the first round of compounds against SARS- CoV-2 PLpro through enzymatic assays.

Structure	Compound ID	PLpro IC_{50} (μM)
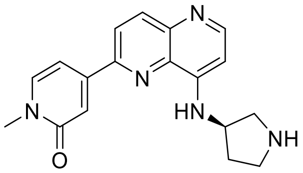	**1**	73.61
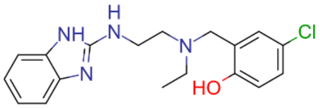	**2**	102.2
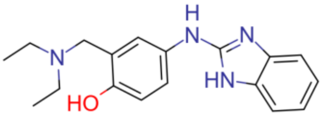	**3**	126.9
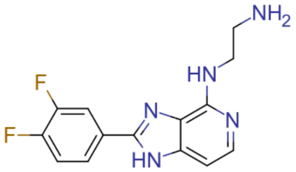	**4**	129.6
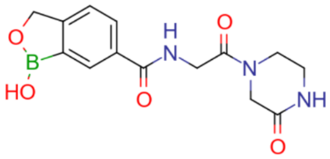	**5**	137.0
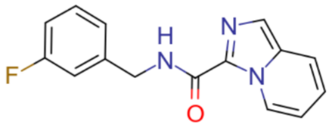	**6**	194.0

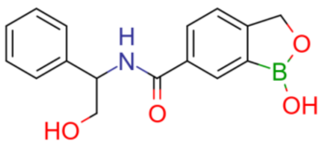	**7**	>270.0
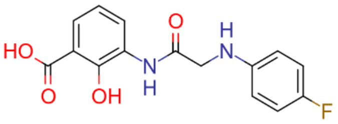	**8**	>276.0

Supplementary Figures

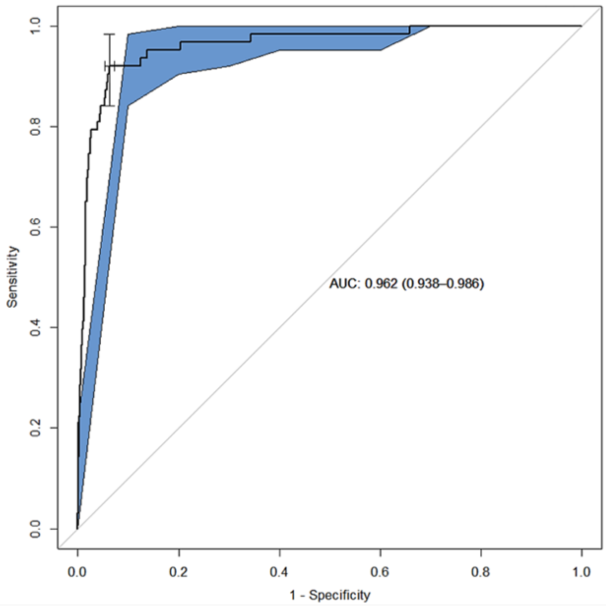

Glide metrics	AUC	TOP 1%		TOP 5%		TOP 10%	
		EF	BEDROC	EF	BEDROC	EF	BEDROC
7LBS	0.962	23.81	0.66	15.87	0.69	9.21	0.77

Figure S1 - AUC curve for docking validation protocol using the structure of PDB ID 7LBS PLpro, as well as docking metrics: AUC, EF, BEDROC for the top 1%, 5% and 10% of ordered list.

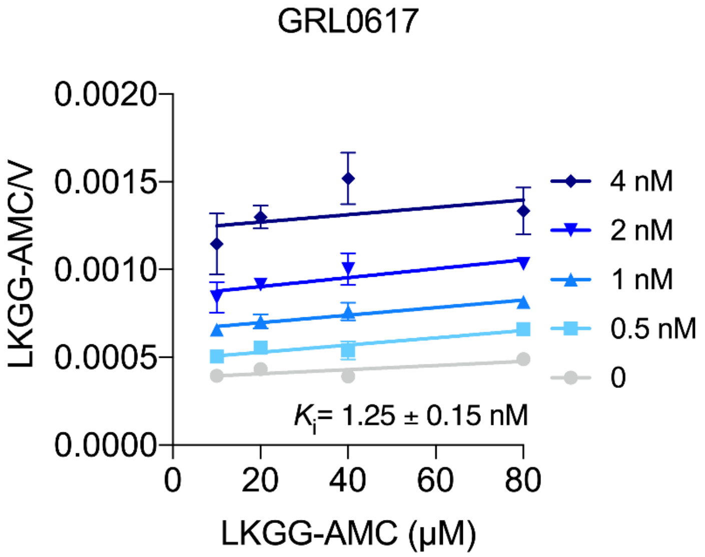

Figure S2 - Hanes-Woolf plot of positive control GRL0617.

Supplementary Results

The analysis of ligand-interactions for the active compounds **1**, **82**, **83**, and **84** (**Figure S3A-5D**), obtained through the docking calculations, suggested that the presence of the naphthyridine ring is important for the interaction with the aromatic residue Tyr268 of BL-2 Loop. Moreover, an aromatic substituent at R^2, like 1-methyl-2-oxo-1,2-dihydropyridin-4-yl (compound **1**), interacts with the hydrophobic site (Pro248 and Pro249) of PLpro and with Tyr268 by a π-stacking interaction.

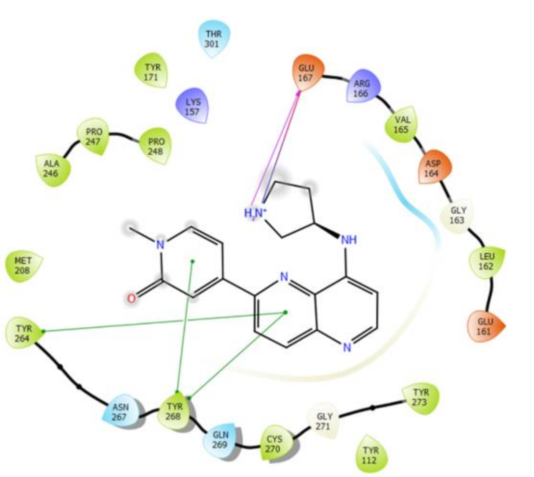

(1)

IC_{50} 73.61μM / Ki 22 μM

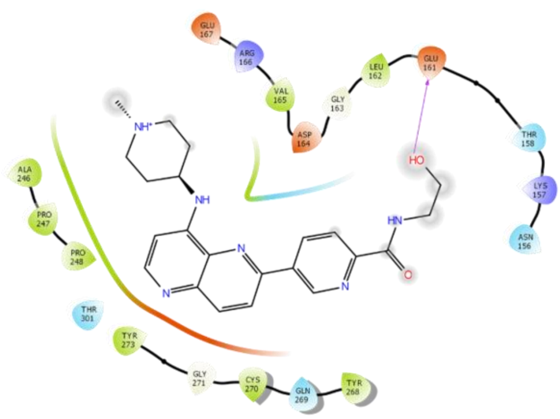

(82)

IC_{50} 79.77 μM

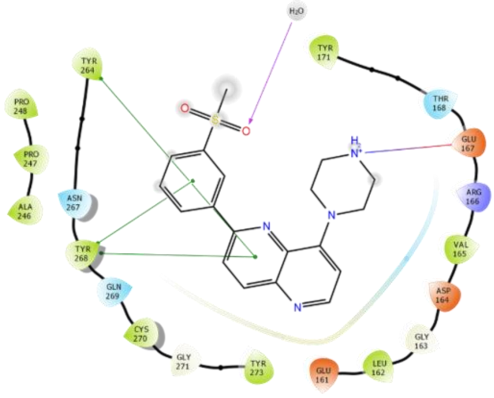

(83)

IC_{50} 54.81 μM

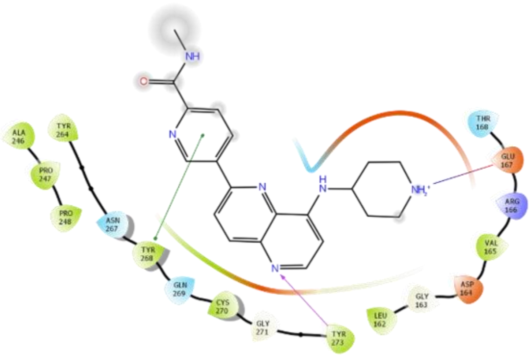

(84)

IC_{50} 39.5 μM

Figure S3. 2D diagram of docked poses of compounds **1, 82, 83 and 84.** Green lines represent π-stacking interactions; red lines represent salt bridges; purple arrows represent hydrogen bonds. The regions of the ligand that are exposed to the solvent are represented with a gray shadow.

Comparing the naphthyridine series with the naphthalene series (GRL0617 and XR8-24 derivatives) reveals that naphthyridines have the ability to engage in stacking interactions with the aromatic residues of the BL-2 loop. In contrast, the naphthalene series interacts with the hydrophobic site, specifically with Pro247 and Pro248. In compound **82** (IC_{50} = 79.77 µM**)**, the presence of a pyridine and carboxamide group at R^1 renders the molecule rigid, disrupting planarity for effective π-stacking interactions with Tyr268 of the BL-2 Loop (**Fig. S3**), reducing the PLpro activity. Meanwhile, the methylpiperidine group at R^2 primarily contributes to hydrophobic interactions, resulting in reduced inhibitory activity. For **compound 83** (IC_{50} = 54.81 µM), the methylsulfonylphenyl group at R^2 has a detrimental effect on activity, reducing hydrophobic interactions and increasing solvent exposure. Conversely, the piperazine ring at R^1 forms a salt bridge with Glu167 (**Fig. S3**), a crucial residue for PLpro activity. **Compound 84** (IC_{50} = 39.50 µM), presents the piperidine at R^1 that maintains a salt bridge with Glu167, while the pyridine-2-carboxamide at R^2 alters ring conformation, leading to a loss of interactions with Tyr268 (**Fig. S3**).

Compounds characterization

LC purity traces were performed using one of the following methods:

Method 1: Using a Kinetex 2.6 µM C-18 column, 2 µL injection volume, flow 0.7 mL/min; gradient: 15-100% B in 1.2 min (hold 3.3 min), 100-15% in 0.3 min (hold 1.2 min) (Mobile phase A: 10 mM buffer (Ammonium acetate/acetic acid) in H2O and Mobile phase B: 10 mM buffer (Ammonium acetate/acetic acid) in Methanol).

Method 2: Using a Kinetex 1.7 µM C-18 column, 1 µL injection volume, flow 1.2 mL/min; gradient: 5-100% B in 1.5 min (hold 0.4 min), 100-5% in 0.3 min (hold 0.5 min) (Mobile phase A: 0.1% formic acid in H2O and Mobile phase B: 0.1% formic acid in Acetonitrile).

Compound 1

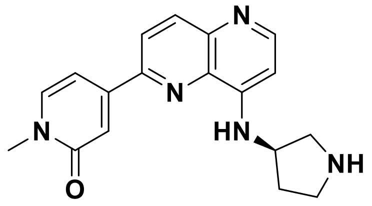

To a solution of tert-butyl (3R)-3-[[6-(1-methyl-2-oxopyridin-4-yl)-1,5-naphthyridin-4-yl]amino]pyrrolidine-1-carboxylate (90.mg, 0.21mmol) in methanol (4mL) was added Hydrogen chloride solution (10.mL, 287.99mmol). The resultant mixture was stirred at 25°C for 18 h. After this time, LC-MS showed formation of the desired compound. To this end, the reaction mixture was concentrated *in vacuo*, the residue was taken up with DCM and adsorbed on isolute. Purification was achieved on Reverse-phase Combi Flash using 0.1% TFA-MeOH. The product fractions were combined, concentrated, frezze dried and free based with Amberlyst, filtetred and dried to furnish 1-methyl-4-[8-[[(3R)-pyrrolidin-3-yl]amino]-1,5-naphthyridin-2-yl]pyridin-2-one (39.58mg,0.1195mmol, 55.947% yield) as a pale-brown compound.

LC-MS: t_R = 0.398 min (method 1, purity 97%); *m/z* = 322.1 $[M+H]^+$ (anal calcd. for $C_{18}H_{19}N_5O$: m/z = 321.1).

^{1}H NMR (600 MHz, DMSO-d_6) δ 9.99 (t, *J* = 11.8 Hz, 1H), 9.66 (s, 1H), 8.68 (d, *J* = 6.9 Hz, 1H), 8.61 (d, *J* = 9.0 Hz, 1H), 8.55 (dd, *J* = 9.0, 2.8 Hz, 1H), 7.84 (d, *J* = 7.1 Hz, 1H), 7.53 (d, *J* = 7.0 Hz, 1H), 7.41 (s, 1H), 7.15 (d, *J* = 7.0 Hz, 1H), 4.91 – 4.65 (m, 1H), 3.55 (d, *J* = 9.2 Hz, 1H), 3.48 (s, 4H), 2.20 (tdd, *J* = 13.4, 11.0, 6.3 Hz, 1H). Note: *Pyrrolidine protons obscured with DMSO-H_2O peak. See the spectrum with TFA below.*

^{1}H NMR (400 MHz, DMSO-d_6 + TFA*)* δ 9.88 (d, *J* = 9.2 Hz, 1H), 9.57 (s, 1H), 9.28 (d, *J* = 8.2 Hz, 1H), 7.89 (d, *J* = 7.1 Hz, 1H), 7.53 (dd, *J* = 7.1, 2.1 Hz, 1H), 7.48 (d, *J* = 2.0 Hz, 1H), 7.22 (d, *J* = 7.1 Hz, 1H), 4.83 (q, *J* = 6.5 Hz, 1H), 3.60 (dq, *J* = 10.9, 5.9 Hz, 2H), 3.52 (s, 3H), 3.30 (dt, *J* = 12.7, 6.7 Hz, 1H), 2.24 (dt, *J* = 11.6, 4.3 Hz, 1H).

Naphthyridine series (round 2)

Compound 82

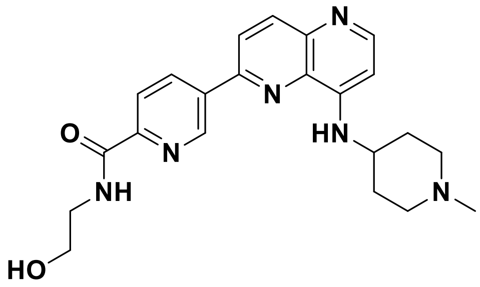

To a solution of 5-(8-chloro-1,5-naphthyridin-2-yl)-N-(2-hydroxyethyl)pyridine-2-carboxamide (150.mg, 0.42 mmol), in 1,4-dioxane (4 mL) was added 4-Amino-1-methylpiperidine (62.99mg, 0.55 mmol), Cesium carbonate (276.51mg, 0.85 mmol), Palladium(II) acetate (9.53mg, 0.04 mmol) and [1-(2-diphenylphosphanylnaphthalen-1-yl)naphthalen-2-yl]-diphenylphosphane (13.21mg, 0.02 mmol) in that order. The solution was degassed by bubbling nitrogen through the mixture. The reaction was heated at 115 °C until full conversion of the starting reagent (3 h).
The reaction was cooled to room temperature, diluted with ETOAc, and adsorbed on to Isolute. The crude was purified by reverse phase (C18) column chromatography eluting a gradient of acetonitrile in water on a Biotage Isolera One Flush Instrument. The desired fractions were concentrated to dryness in vacuo to obtain the product, N-(2-hydroxyethyl)-5-[8-[(1-methylpiperidin-4-yl)amino]-1,5-naphthyridin-2-yl]pyridine-2-carboxamide (49 mg, 0.1205 mmol, 28.409 %) as a lemon-green viscous oil, which taken up with diethyl ether and pentane mixtures precipitated as yellow solid.

LC-MS: t_R = 0.151 min (method 2, purity 100%); *m/z* = 407.2 $[M+H]^+$ (anal calcd. for $C_{22}H_{26}N_6O_2$: m/z = 406.2).

^{1}H NMR (300 MHz, DMSO-d_6) δ 9.62 (d, *J* = 2.2 Hz, 1H), 8.98 (dd, *J* = 8.2, 2.2 Hz, 1H), 8.78 (t, *J* = 5.8 Hz, 1H), 8.51 – 8.41 (m, 2H), 8.30 (d, *J* = 8.8 Hz, 1H), 8.18 (d, *J* = 8.2 Hz, 1H), 7.27 (d, *J* = 8.3 Hz, 1H), 6.78 (d, *J* = 5.5 Hz, 1H), 3.72 – 3.60 (m, 1H), 3.60 – 3.52 (m, 2H), 3.49 – 3.40 (m, 2H), 3.03 – 2.91 (m, 2H), 2.40 – 2.29 (m, 5H), 2.08 – 1.95 (m, 2H), 1.92 – 1.75 (m, 2H).

Compound 83 (Compound 20 in Kandepedu 2018)

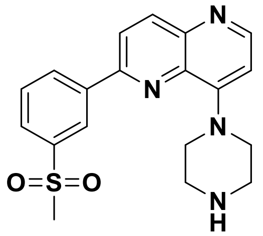

Compound 83 was prepared using General procedure 6 in Kandepedu 2018. Yield 36%.

LC-MS: t_R = 0.485 min (method 1, purity 98%); *m/z* = 369.1 $[M+H]^+$ (anal calcd. for $C_{19}H_{20}N_4O_2S$: m/z = 368.1).

^{1}H NMR (300 MHz, DMSO-*d*$_6$) δ = 8.84 (d, *J* = 1.9 Hz, 1H), 8.59 (dd, *J* = 9.4, 6.4 Hz, 2H), 8.49 – 8.34 (m, 2H), 8.11 – 8.00 (m, 1H), 7.86 (t, *J* = 7.8 Hz, 1H), 6.99 (d, *J* = 5.3 Hz, 1H), 3.69 (dd, *J* = 6.2, 3.5 Hz, 4H), 3.03 (dd, *J* = 6.1, 3.5 Hz, 4H), 2.65 (s, 3H).

Compound 84

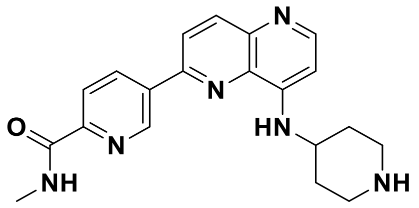

To a 50 mL round-bottom flask was added tert-butyl 4-[[6-[6-(methylcarbamoyl)pyridin-3-yl]-1,5-naphthyridin-4-yl]amino]piperidine-1-carboxylate (120, mmol) dissolved in 3 ml DCM, followed by addition of Hydrogen chloride solution (10500, mmol). The mixture was stirred at room temperature for 18 h. After this time, LCMS indicated that the starting material had been consumed, and the desired product mass was observed. The reaction mixture was then concentrated to dryness and then adsorbed on Isolute.
Purification was achieved on Reverse phase ISCO Combi Flash using positive gradient of 0.1%TFA/H2O-MeOH on a C18 12g silicycle. The product fractions were combined and concentrated, excess solvent was removed on freeze-drier affording a yellow solid which was stirred in Amberlyst and it then dried to furnish N-methyl-5-[8-(piperidin-4-ylamino)-1,5-naphthyridin-2-yl]pyridine-2-carboxamide (42.65 mg, 0.1177 mmol, 45.359 %) as a yellow solid product.

LC-MS: t_R = 0.674 min (method 1, purity 100%); *m/z* = 363.2 $[M+H]^+$ (anal calcd. for $C_{20}H_{22}N_6O$: m/z = 362.2).

^{1}H NMR (300 MHz, DMSO-*d*$_6$) δ 9.67 (d, *J* = 2.2 Hz, 1H), 9.44 – 9.35 (m, 1H), 9.31 (d, *J* = 8.7 Hz, 1H), 9.07 (dd, *J* = 8.3, 2.2 Hz, 1H), 8.89 (q, *J* = 4.7 Hz, 1H), 8.79 (d, *J* = 9.0 Hz, 1H), 8.72 (d, *J* = 7.1 Hz, 1H), 8.62 (d, *J* = 8.9 Hz, 1H), 8.21 (d, *J* = 8.2 Hz, 1H), 7.30 (d, *J* = 7.1 Hz, 1H), 4.27 (s, 1H), 3.09 (d, *J* = 12.9 Hz, 2H), 2.88 (d, *J* = 4.8 Hz, 3H), 2.14 (d, *J* = 10.0 Hz, 5H). 1*H Obsurred under the DMSO-H_2O peak.*

^{13}C NMR (151 MHz, DMSO-*d*$_6$) δ 164.45, 154.81, 152.77, 151.42, 148.48, 143.60, 137.22, 134.73, 134.36, 132.35, 130.63, 126.42, 122.21, 101.15, 48.93, 42.65, 27.86, 26.63.

Compound 85

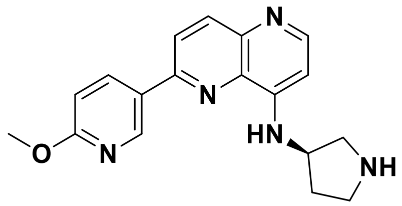

To a flask containing tert-butyl (3R)-3-[[6-(6-methoxypyridin-3-yl)-1,5-naphthyridin-4-yl]amino]pyrrolidine-1-carboxylate (120.mg, 0.28 mmol)was added DCM (3 mL) and Hydrogen chloride solution (3.mL, 86.4 mmol) (4M in dioxane). The reaction mixture was stirred for 18 h at ambient temperature. The excess HCl and DCM were removed under reduced pressure and the resulting residue taken up in MeOH and adsorbed onto isolute. Purification was carried out using a Teledyne ISCO CombiFlash system eluting a reverse phase solvent gradient of MeOH in 0.1% TFA/Water on a 13 g C18 column. The fractions containing the desired product were combined and the solvents removed on the freeze drier. The resulting residue was taken up in MeOH/DCM and amberlyst added to neutralise the salt. The amberlyst was filtered off and the filtrate concentrated to dryness. The compound did not become solid and free base also contained some impurity in LCMS. It was again treated with HCl-Dioxane to form HCl salt followed by purification using a Teledyne ISCO CombiFlash system eluting a reverse phase solvent gradient of MeOH in 0.1% TFA/Water on a 13 g C18 column. The solvent was evaporated and the residue was washed with pentane to afford 6-(6-methoxypyridin-3-yl)-N-[(3R)-pyrrolidin-3-yl]-1,5-naphthyridin-4-amine (42 mg, 0.1281 mmol, 45.903 %) as a light yellow solid TFA salt.

LC-MS: t_R = 0.201 min (method 1, purity 97%); *m/z* = 322.2 $[M+H]^+$ (anal calcd. for $C_{18}H_{19}N_5O$: m/z = 321.2).

^{1}H NMR (400 MHz, DMSO-*d*$_6$) δ 9.37 (s, 1H), 9.32 (d, *J* = 2.5 Hz, 1H), 9.22 (s, 1H), 9.02 (d, *J* = 7.8 Hz, 1H), 8.82 (dd, *J* = 8.7, 2.5 Hz, 1H), 8.73 (d, *J* = 6.9 Hz, 1H), 8.65 (d, *J* = 9.0 Hz, 1H), 8.45 (d, *J* = 9.0 Hz, 1H), 7.20 (d, *J* = 7.0 Hz, 1H), 7.04 (d, *J* = 8.7 Hz, 1H), 4.81 (d, *J* = 6.2 Hz, 1H), 3.98 (s, 3H), 3.68 (d, *J* = 13.0 Hz, 2H), 3.62 – 3.43 (m, 2H), 3.36 (s, 1H), 2.26 (dq, *J* = 14.8, 7.8 Hz, 1H).

^{13}C NMR (101 MHz, DMSO-*d*$_6$) δ 165.45, 158.93, 158.60, 155.02, 154.07, 147.90, 143.53, 138.86, 133.49, 132.10, 130.54, 126.58, 125.71, 118.49, 115.54, 111.21, 101.34, 54.10, 52.28, 49.00, 44.68, 30.27. (CF3 quartet was observed)

^{19}F NMR (377 MHz, DMSO) δ -73.84.

Compound 86 (Compound 31 in Kandepedu 2018)

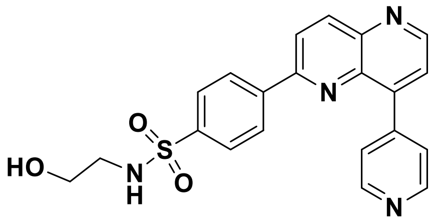

Compound **86** was prepared using General procedure 5 in Kandepedu 2018. Yield 29%.

LC-MS: t_R = 3.396 min (method 1, purity 99%); *m/z* = 401.1 $[M+H]^+$ (anal calcd. for $C_{21}H_{18}N_4O_3S$: m/z = 406.1).

^{1}H NMR (300 MHz, DMSO-d_6) δ = 9.14 (d, 1H, *J* = 4.4 Hz), 8.86-8.78 (m, 2H), 8.67 (d, 1H, *J* = 8.9 Hz), 8.56 (d, 1H, *J* = 8.9 Hz), 8.41 (d, 2H, *J* = 8.4 Hz), 8.03-7.87 (m, 5H), 4.68 (br s, 1H), 4.02 (br s, 1H), 3.38 (d, 1H, *J* = 6.2 Hz), 2.85 (t, 2H, *J* = 6.3 Hz).

Compound 87

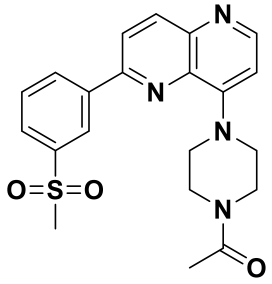

Add 8-chloro-2-(3-methylsulfonylphenyl)-1,5-naphthyridine (100.mg, 0.31mmol), Cesium carbonate (204.42mg, 0.63mmol) *N,N*-dimethylformamide (2mL) and 1-Acetylpiperazine (100.52mg, 0.78mmol) to a 7 mL reaction vial. Heat the mixture to 110°C and stir for 18 h. LCMS confirmed that the desired product was obtained. While there was trace amounts of starting material remaining, the reaction was stopped and the DMF removed under reduced pressure using toluene as an azeotrope. The remaining residue was diluted with EtOAc and washed with 3 x 15 mL water and 1 x 20 mL brine. The organic fraction was dried over anhydrous magnesium sulphate, filtered and the filtrate adsorbed onto silica gel.

Purification was carried out using a Teledyne ISCO CombiFlash system, eluting with a gradient of 0 - 15% MeOH in DCM over 15 min. The product elutes at roughly 10 - 11% MeOH in DCM. The fractions containing the desired product were combined and the solvent removed under reduced pressure to afford 1-[4-[6-(3-methylsulfonylphenyl)-1,5-naphthyridin-4-yl]piperazin-1-yl]ethanone (56.6mg,0.1379mmol, 43.955% yield) as a pale yellow powder.

LC-MS: t_R = 0.657 min (method 1, purity 100%); *m/z* = 411.1 $[M+H]^+$ (anal calcd. for $C_{21}H_{22}N_4O_3S$: m/z = 410.1).

^{1}H NMR (300 MHz, DMSO-d_6) δ 8.83 (s, 1H), 8.65 (d, *J* = 5.2 Hz, 1H), 8.59 (d, *J* = 7.9 Hz, 1H), 8.52 – 8.39 (m, 2H), 8.06 (d, *J* = 7.8 Hz, 1H), 7.86 (t, *J* = 7.8 Hz, 1H), 7.03 (d, *J* = 5.2 Hz, 1H), 3.79 (d, *J* = 11.2 Hz, 8H), 3.32 (s, 3H), 2.09 (s, 3H).

^{13}C NMR (151 MHz, DMSO-d_6) δ 168.64, 153.52, 151.74, 150.68, 144.13, 142.02, 139.31, 139.08, 137.25, 131.72, 130.48, 127.89, 125.47, 121.40, 109.56, 50.28, 49.91, 45.72, 43.70, 40.92, 39.52, 21.38.

Compound 88

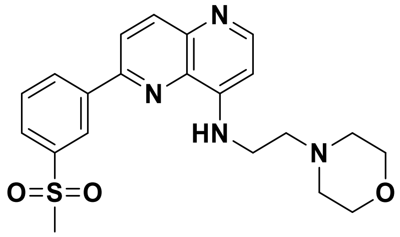

To a solution of 8-chloro-2-(3-methylsulfonylphenyl)-1,5-naphthyridine (100.mg, 0.31mmol) in *N,N*-dimethylformamide (2mL) was added Cesium carbonate (306.63mg, 0.94mmol) as the base, followed by the addition of 4-(2-Aminoethyl)morpholine (0.16mL, 1.25mmol). The resultant mixture was heated at 110°C in a sealed tube for 18 h. After this time, LC-MS showed formation of the desired compound and no traces of the starting material. Excess of DMF was removed on in vacuo with the help of toluene, the residue was extracted with 2x100 ml of ethyl acetate and 200 ml of water. The combined organic extracts were dried over MgSO4, filtered and excess solvent removed on rotary evaporator. The crude compound was adsorbed on silica and purified on ISCO CombiFlash column, using 24g Silicycle and eluting with 90% EtOAc and 10% MeOH. The test tube containing the desired compound were combined and concentrated in vacuo to give 6-(3-methylsulfonylphenyl)-N-(2-morpholin-4-ylethyl)-1,5-naphthyridin-4-amine (70mg,0.1646mmol, 52.472% yield) as a yellow solid compound.

LC-MS: t_R = 0.399 min (method 1, purity 100%); *m/z* = 412.1 $[M+H]^+$ (anal calcd. for $C_{21}H_{24}N_4O_3S$: m/z = 413.1).

^{1}H NMR (300 MHz, DMSO-d_6) δ 8.74 (d, *J* = 8.7 Hz, 2H), 8.48 (d, *J* = 5.3 Hz, 1H), 8.43 (d, *J* = 8.9 Hz, 1H), 8.31 (d, *J* = 8.8 Hz, 1H), 8.06 (d, *J* = 7.7 Hz, 1H), 7.85 (t, *J* = 7.7 Hz, 1H), 7.53 (s, 1H), 6.71 (d, *J* = 5.3 Hz, 1H), 3.65 (t, *J* = 4.5 Hz, 4H), 3.55 – 3.45 (m, 2H), 3.35 (s, 3H), 2.70 (t, *J* = 6.4 Hz, 2H).

^{13}C NMR (151 MHz, DMSO-d_6) δ 152.45, 151.19, 150.18, 142.81, 142.28, 139.72, 138.53, 134.74, 132.41, 130.46, 128.04, 125.56, 122.46, 100.94, 66.80, 56.50, 53.59, 43.86, 38.94.

Compound 89

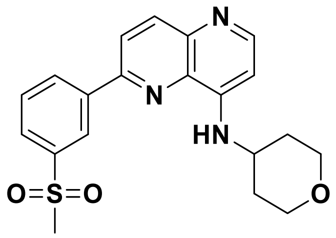

To a solution of 8-chloro-2-(3-methylsulfonylphenyl)-1,5-naphthyridine (100.mg, 0.31mmol) in *N,N*-dimethylformamide (2mL) was added Cesium carbonate (306.63mg, 0.94mmol), followed by the addition of 4-Aminotetrahydropyran (0.13mL, 1.25mmol). The resultant mixture was heated at 110°C in a sealed tube for 18 h. After this time, LC-MS showed formation of the desired compound. DMF was removed on *in vacuo* under reduced pressure. The residue was extracted with 2 x 100 ml of ethyl acetate and 200 ml of water. The combined organic extracts were dried over $MgSO_4$, filtered and excess solvent removed on rotary evaporator. The crude compound was adsorbed on silica and purified on ISCO CombiFlash column, using 24g Silicycle and eluting with 90% EtOAc and 10% MeOH. The product fractions were combined and concentrated *in vacuo* to give a yellow product (impure) which was then recrystallised with DCM/Hexanes to furnish 6-(3-methylsulfonylphenyl)-N-(oxan-4-yl)-1,5-naphthyridin-4-amine (9.11mg,0.0238mmol, 7.5734% yield) as a yellow solid compound.

LC-MS: t_R = 0.682 min (method 1, purity 100%); *m/z* = 384.1 $[M+H]^+$ (anal calcd. for $C_{20}H_{21}N_3O_3S$: m/z = 383.1).

^{1}H NMR (300 MHz, DMSO-d_6) δ 8.79 (dt, *J* = 8.0, 1.4 Hz, 1H), 8.73 (d, *J* = 1.8 Hz, 1H), 8.48 (d, *J* = 5.4 Hz, 1H), 8.42 (d, *J* = 8.9 Hz, 1H), 8.31 (d, *J* = 8.8 Hz, 1H), 8.09 – 8.02 (m, 1H), 7.85 (t, *J* = 7.8 Hz, 1H), 7.10 (d, *J* = 8.4 Hz, 1H), 6.83 (d, *J* = 5.4 Hz, 1H), 4.00 – 3.79 (m, 3H), 3.60 – 3.44 (m, 2H), 3.37 (s, 3H), 2.01 (d, *J* = 10.3 Hz, 2H), 1.75 (qd, *J* = 11.5, 4.3 Hz, 2H). *Note: There is a trace amount of EtOAc*

Compound 90

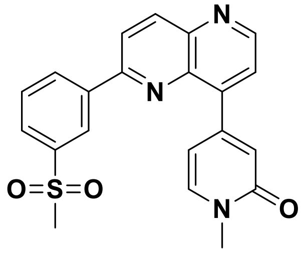

In a 7ml reaction vial, containing 8-chloro-2-(3-methylsulfonylphenyl)-1,5-naphthyridine (100.mg, 0.31mmol), 1-methyl-4-(4,4,5,5-tetramethyl-1,3,2-dioxaborolan-2-yl)pyridin-2-one (147.49mg, 0.63mmol), [1,1'-Bis(diphenylphosphino)-ferrocene] dichloropalladium (II) (22.95mg, 0.03mmol), Cesium carbonate (306.63mg, 0.94mmol) was added 3 ml of dioxane. The resultant mixture was degassed for 5 minutes before adding water (0.5000mL) of degassed water. The resulting mixture was further degassed for 5 minutes. The resultant mixture was heated at 110°C for 18 h. LC-MS

indicated complete consumption of the starting material an formation of the desired product. To this end, the reaction mixture was cooled to room temperature, diljuted with DCM and filtered through celite bed. The residue was redissolved with DCM and adsorbed on silica gel. The slurry was purified on CombiFlash ISCO, eluting with 10%MeOH/ethyl acetate on a 25g silicycle. The test tubes containing the product were combined and concentrated to furnish 1-methyl-4-[6-(3-methylsulfonylphenyl)-1,5-naphthyridin-4-yl]pyridin-2-one (28.15mg,0.0719mmol, 22.92% yield) as a pale-yellow.

LC-MS: t_R = 0.869 min (method 1, purity 100%); m/z = 391.1 $[M + H]^+$ (anal. calcd. For $C_{21}H_{17}N_3O_3S$: m/z = 392.1).

^{1}H NMR (300 MHz, DMSO-d_6) δ 9.09 (d, J = 4.4 Hz, 1H), 8.77 (d, J = 1.8 Hz, 1H), 8.60 (dd, J = 15.5, 6.7 Hz, 3H), 8.07 (d, J = 7.8 Hz, 1H), 7.94 – 7.74 (m, 3H), 6.88 (d, J = 1.8 Hz, 1H), 6.78 (dd, J = 7.0, 1.9 Hz, 1H), 3.55 (s, 3H), 3.34 (s, 3H).

^{13}C NMR (151 MHz, DMSO-d_6) δ 162.12, 155.01, 152.16, 148.35, 144.45, 143.61, 142.35, 140.49, 139.52, 139.32, 138.87, 132.52, 130.68, 128.51, 126.26, 124.62, 122.73, 120.62, 108.16, 43.83, 37.06.

Compound 91

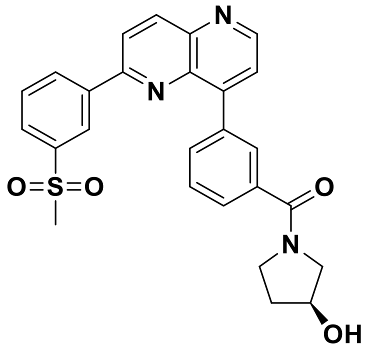

In a 7 ml reaction vessel, 8-chloro-2-(3-methylsulfonylphenyl)-1,5-naphthyridine (100.mg, 0.31mmol), [(3S)-3-hydroxypyrrolidin-1-yl]-[3-(4,4,5,5-tetramethyl-1,3,2-dioxaborolan-2-yl)phenyl]methanone (119.4mg, 0.38mmol), [1,1′-Bis(diphenylphosphino)ferrocene]dichloropalladium(II) (23.02mg, 0.03mmol), Cesium carbonate (306.63mg, 0.94mmol) was placed and degassed using N_2 gas for 5 minutes,. To this was added dry 1,4-dioxane (4mL) via a syringe and the reaction was degassed further for 5 minutes. Deionised water (1mL) was then added to the reaction mixture. The reaction was then heated at 110°C for 18 h. LC-MS indicated complete consumption of the starting material an formation of the desired product. To this end, the reaction mixture was concentrated in vacuo, re-dissolved with DCM and adsorbed on silica gel. Purification was achieved using CombiFlash ISCO, eluting with 10%MeOH/EtOAc, the combined test tubes containing the product were combined and concentrated to afford [(3S)-3-hydroxypyrrolidin-1-yl]-[3-[6-(3-methylsulfonylphenyl)-1,5-naphthyridin-4-yl]phenyl]methanone (15.53mg,0.0328mmol, 10.45% yield) as a pale yellow compound.

LC-MS: t_R = 2.363 min (method 2, purity 100%); m/z = 474.1 $[M + H]^+$ (anal. calcd. for $C_{26}H_{23}N_3O_4S$: m/z = 473.1).

^{1}H NMR (300 MHz, DMSO-d_6) δ 9.09 (d, *J* = 4.4 Hz, 1H), 8.65 (dt, *J* = 28.7, 9.2 Hz, 4H), 8.05 (d, *J* = 8.1 Hz, 3H), 7.94 (d, *J* = 4.5 Hz, 1H), 7.84 (d, *J* = 7.6 Hz, 1H), 7.68 (d, *J* = 8.6 Hz, 2H), 4.94 (d, *J* = 20.2 Hz, 1H), 4.26 (d, *J* = 35.1 Hz, 1H), 3.60 (d, *J* = 10.5 Hz, 1H), 2.08 – 1.61 (m, 2H), 1.21 (d, *J* = 18.5 Hz, 1H), 0.87 (s, 1H), 4H obscurred with DMSO-Water peak.

Compound 92

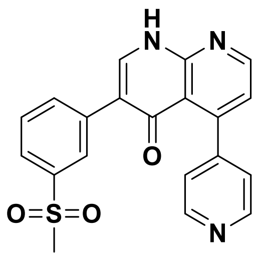

The starting materials 3-Methylsulfonylphenylboronic acid (79.44mg, 0.4mmol), Cesium carbonate (258.82mg, 0.79mmol), 3-bromo-5-pyridin-4-yl-1H-1,8-naphthyridin-4-one (80.mg, 0.26mmol), [1,1'-Bis(diphenylphosphino)-ferrocene] dichloropalladium (II) (19.37mg, 0.03mmol) were placed in a 50 ml rbf and degassed using nitrogen balloon for 5 minutes. To this was then added a degassed 1,4-dioxane (10mL). The resultant mixture was stirred at room temperature for 5 minutes before the addition of water (1mL) which initiated the Suzuki coupling reaction. The dark brown mixture was left to stir at 100°C for 18 h. After this time, LC-MC showed total consumption of the starting material and formation of the desired product. Excess dioxane was removed on rotary evaporator, redissolved in DCM , passed through celite bed and adsorbed on silica. Purification was achieved on CombiFlash ISCO, eluting with 10%MeOH/ethyl acetate. The product fractions were combined and concentrated *in vacuo* to give a yellow solid product, which was then recrystallised with Hexane/ethyl acetate to give 3-(3-methylsulfonylphenyl)-5-pyridin-4-yl-1H-1,8-naphthyridin-4-one (8.05mg,0.0207mmol, 7.8135% yield) as a yellow solid product.

LC-MS: t_R = 0.141 min (method 1, purity 95%); *m/z* = 378.1 $[M+H]^+$ (anal calcd. for $C_{20}H_{15}N_3O_3S$: m/z = 377.1).

^{1}H NMR (300 MHz, DMSO-d_6**)** δ 9.20 (d, *J* = 7.4 Hz, 1H), 8.80 (d, *J* = 8.2 Hz, 3H), 8.28 (s, 1H), 8.18 (d, *J* = 8.0 Hz, 2H), 8.06 – 7.87 (m, 5H), 3.26 (s, 3H). *Please note the sulphonyl methyl obscured under the DMSO-H_2O peak.*

^{13}C NMR (151 MHz, DMSO-d_6**)** δ 156.22, 154.30, 151.54, 151.20, 145.78, 142.76, 140.12, 139.88, 129.18, 128.97, 127.33, 123.86, 121.89, 115.57, 114.14, 44.18

Compound 93

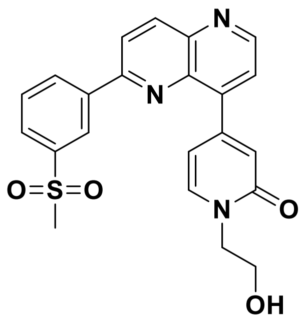

1,4-dioxane (4mL) was added to a mixture of 8-chloro-2-(3-methylsulfonylphenyl)-1,5-naphthyridine (50.mg, 0.16mmol), Tris(dibenzylideneacetone)dipalladium(0) (14.36mg, 0.02mmol), Tricyclohexylphosphine (10.56mg, 0.04mmol), Potassium phosphate tribasic (99.88mg, 0.47mmol), [1-(2-hydroxyethyl)-2-oxopyridin-4-yl]boronic acid (57.4mg, 0.31mmol). The resultant solution was then degassed for few minutes before the addition of water (1mL). The mixture was then degassed for further 5 minutes before being placed in a microwave. The reaction mixture was irradiated at 120°C for 15 min, 200W (Dynamic mode). LC-MS analysis showed formation of the desired product. To this end, the reaction mixture was cooled to room temperature, diluted with DCM and filtered through celite bed. The residue was redissolved with DCM and adsorbed on silica gel. The resulting slurry was purified on CombiFlash ISCO, eluting with 10%MeOH/ethyl acetate using 25g silicycle. The test tube containing the product were combined and concentrated *in vacuo* to give a light-brown solid product, which was then recrystallised with Hexane/ethyl acetate to give 1-(2-hydroxyethyl)-4-[6-(3-methylsulfonylphenyl)-1,5-naphthyridin-4-yl]pyridin-2-one (21.56mg,0.0512mmol, 32.61% yield) as a Brown solid.

LC-MS: t_R = 0.810 min (method 1, purity 99%); m/z = 422.1 $[M + H]^+$ (anal. calcd. For $C_{22}H_{19}N_3O_4S$: m/z = 421.1).

^{1}H NMR (300 MHz, DMSO-d_6) δ 9.10 (d, J = 4.4 Hz, 1H), 8.78 (t, J = 1.8 Hz, 1H), 8.68 – 8.53 (m, 3H), 8.07 (dt, J = 8.1, 1.2 Hz, 1H), 7.92 (d, J = 4.4 Hz, 1H), 7.85 (t, J = 7.8 Hz, 1H), 7.75 (d, J = 7.0 Hz, 1H), 6.89 (d, J = 1.9 Hz, 1H), 6.78 (dd, J = 7.0, 2.0 Hz, 1H), 4.95 (t, J = 5.3 Hz, 1H), 4.06 (td, J = 5.3, 2.2 Hz, 2H), 3.73 (q, J = 5.5 Hz, 2H), 3.33 (s, 3H).

^{13}C NMR (101 MHz, DMSO-d_6) δ 161.79, 155.04, 152.20, 148.32, 144.49, 143.66, 142.44, 140.54, 139.59, 139.35, 139.13, 132.55, 130.69, 128.47, 126.34, 124.58, 122.73, 120.92, 107.79, 59.30, 51.64, 43.88.

Compound 94

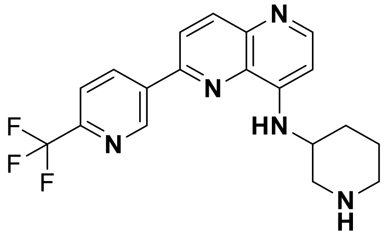

Hydrogen chloride solution (6.62mL, 26.48mmol) was added to a flask containing tert-butyl 3-[[6-[6-(trifluoromethyl)pyridin-3-yl]-1,5-naphthyridin-4-yl]amino]piperidine-1-carboxylate (177.mg, 0.37mmol). The reaction mixture was then left to stir at room temperature overnight (18 h), LCMS indicating full conversion of the starting material to the desired product after this time.

The reaction mixture was concentrated under reduced pressure and the resulting residue diluted with 15mL 10% MeOH in DCM. Amberlyst 21 was added and the suspension stirred for 2 h to neutralize to pH ~ 7. The Amberlyst was filtered off and the filtrate concentrated under reduced pressure. The residue was adsorbed onto isolute and purified using a Teledyne ISCO CombiFlash system eluting a reverse phase solvent gradient of Methanol in 0.1% TFA/Water on a 12g C18 column. The fractions containing the desired product were combined and the solvent removed under reduced pressure (then freeze dried overnight). The residue was taken up in 10mL 10% MeOH/DCM and a small scoop of Amberlyst was added to neutralise the excess TFA. The Amberlyst was filtered off and the filtrate concentrated to dryness to afford N-piperidin-3-yl-6-[6-(trifluoromethyl)pyridin-3-yl]-1,5-naphthyridin-4-amine (74mg,0.1962mmol, 52.49% yield) as a yellow solid.

LC-MS: t_R = 0.556 min (method 1, purity 99%); m/z = 374.2 $[M + H]^+$ (anal. calcd. For $C_{19}H_{18}F_3N_5$: m/z = 373.2).

^{1}H NMR (400 MHz, DMSO-d_6) δ 9.73 (s, 1H), 8.94 (d, J = 9.0 Hz, 1H), 8.47 (d, J = 5.3 Hz, 1H), 8.43 (d, J = 8.9 Hz, 1H), 8.31 (d, J = 8.8 Hz, 1H), 8.07 (d, J = 8.2 Hz, 1H), 7.31 (d, J = 8.7 Hz, 1H), 6.74 (d, J = 5.4 Hz, 1H), 3.74 – 3.61 (m, 1H), 3.08 (dd, J = 11.9, 2.8 Hz, 1H), 2.87 – 2.76 (m, 1H), 2.74 – 2.66 (m, 1H), 2.65 – 2.56 (m, 1H), 1.98 – 1.89 (m, 1H), 1.82 – 1.72 (m, 1H), 1.72 – 1.63 (m, 1H), 1.57 – 1.45 (m, 1H).

^{13}C NMR (101 MHz, DMSO-d_6) δ 152.41, 152.27, 148.99, 148.85, 148.73, 142.78, 142.72, 138.11, 136.53, 136.37, 134.49, 122.23, 120.70, 100.55, 50.69, 48.46, 45.89, 29.48, 24.46.

Compound 95

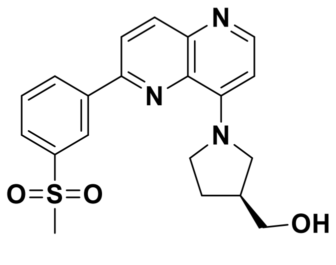

8-chloro-2-(3-methylsulfonylphenyl)-1,5-naphthyridine (100.mg, 0.31mmol), Cesium carbonate (204.42mg, 0.63mmol) *N*,*N*-dimethylformamide (2.5mL) and (S)-Pyrrolidin-3-ylmethanol (95.19mg, 0.94mmol) were added to a 7 mL reaction vial. The mixture was heated to 110°C and stir for 18 h. LCMS confirmed that the desired product was obtained. The reaction mixture was cooled to room temperature and the DMF removed under reduced pressure (using toluene as an azeotrope).

The residue was triturated with EtOAc then n-pentane and dried under high vacuum to give [(3S)-1-[6-(3-methylsulfonylphenyl)-1,5-naphthyridin-4-yl]pyrrolidin-3-yl]methanol (81mg,0.2007mmol, 63.97% yield) as a beige powder.

LC-MS: t_R = 0.693 min (method 1, purity 100%); *m/z* = 384.1 $[M+H]^+$ (anal calcd. for $C_{20}H_{21}N_3O_3S$: m/z = 383.1).

^{1}H NMR (300 MHz, DMSO-d_6) δ 8.79 (s, 1H), 8.59 (d, *J* = 7.9 Hz, 1H), 8.51 – 8.40 (m, 2H), 8.33 (d, *J* = 8.9 Hz, 1H), 8.08 (d, *J* = 7.8 Hz, 1H), 7.90 (t, *J* = 7.8 Hz, 1H), 6.67 (d, *J* = 5.5 Hz, 1H), 4.09 (s, 4H), 3.85 (s, 1H), 3.60 (d, *J* = 6.6 Hz, 2H), 2.26 – 2.13 (m, 1H), 1.97 – 1.85 (m, 1H).

^{13}C NMR (151 MHz, DMSO-d_6) δ 168.11, 154.26, 151.67, 145.94, 143.36, 141.84, 140.33, 139.05, 138.88, 137.42, 137.23, 131.95, 130.69, 130.23, 128.02, 126.78, 125.80, 124.55, 121.89, 68.79, 56.67, 52.04, 43.45, 40.48, 39.52, 20.45.

Compound 96 (Compound 16 in Dziwornu 2024)

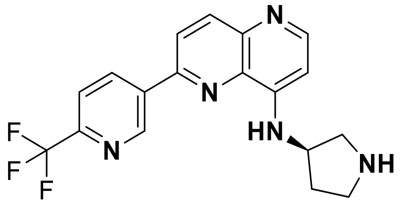

To a solution of tert-butyl rac-(3R)-3-[[6-[6-(trifluoromethyl)pyridin-3-yl]-1,5-naphthyridin-4-yl]amino]pyrrolidine-1-carboxylate (80.mg, 0.17mmol) in methanol (4mL) was added 4M Hydrogen chloride solution (11.77mg, 0.32mmol) The resultant mixture was stirred at room temperature for 18 h. After this time, LC-MS showed formation of the desired compound. To this end, excess of HCl was removed in vacuo. The residue was re-dissolved with DCM and adsorbed on isolute, purified on Reverse phase using 0.1%TFA/Methanol. The fractions containing the product were combined, concentrated and freeze dried to furnish a yellow compound which was free-based with amberlyst to give N-[rac-(3R)-pyrrolidin-3-yl]-6-[6-(trifluoromethyl)pyridin-3-yl]-1,5-naphthyridin-4-amine (22.25mg,0.0594mmol, 34.14% yield) as a yellow solid.

LC-MS: t_R = 0.308 min (method 1, purity 99%); m/z = 360.1 $[M + H]^+$ (anal. calcd. for C18H16F3N5: m/z = 359.1).

^{1}H NMR (300 MHz, DMSO-d_6) δ 9.80 (d, J = 2.1 Hz, 1H), 9.00 (dd, J = 8.2, 2.2 Hz, 1H), 8.53–8.41 (m, 2H), 8.33 (d, J = 8.8 Hz, 1H), 8.06 (d, J = 8.2 Hz, 1H), 7.28 (d, J = 7.2 Hz, 1H), 6.74 (d, J = 5.4 Hz, 1H), 4.14 (dq, J = 6.9, 3.6, 2.9 Hz, 1H), 3.22–3.09 (m, 1H), 3.11–2.96 (m, 1H), 2.96– 2.76 (m, 2H), 2.19 (tt, J = 13.9, 7.1 Hz, 1H), 1.84 (td, J = 12.4, 6.2 Hz, 1H).

^{13}C NMR (101 MHz, DMSO-d_6) δ 158.86, 155.32, 152.46, 150.12, 144.36, 137.78, 135.73, 134.29, 132.34, 131.03, 126.84, 121.25, 117.95, 115.03, 101.62, 52.41, 48.95, 44.68, 30.24.

Compound 97

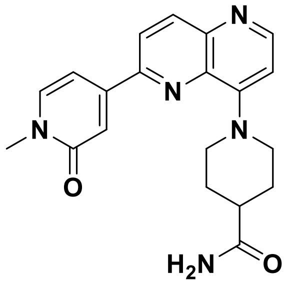

To a solution of 4-(8-chloro-1,5-naphthyridin-2-yl)-1-methylpyridin-2-one (80.mg, 0.29mmol) in *N,N*-dimethylacetamide (4mL) was added Cesium carbonate (287.81mg, 0.88mmol), followed by the addition of Isonipecotamide (0.15mL, 1.18mmol). The resultant mixture was heated at 110°C in a sealed tube for 18 h. After this time, LC-MS showed formation of the desired compound. After cooling to room temperature, the reaction mixture was diluted with DCM and concentrated in vacuo. The residue was adsorbed on silica and purified on ISCO CombiFlash column, using 12g Silicycle and eluting with 90% EtOAc and 10% MeOH. The product fractions were combined and concentrated under reduced pressure to afrord 1-[6-(1-methyl-2-oxopyridin-4-yl)-1,5-naphthyridin-4-yl]piperidine-4-carboxamide (41.04mg,0.1095mmol, 37.203% yield) as a yellow solid compound, which was dried further under vacuum oven.

LC-MS: t_R = 1.050 min (method 1, purity 97%); *m/z* = 364.2 $[M+H]^+$ (anal calcd. for $C_{20}H_{21}N_5O_2$: m/z = 363.2).

^{1}H NMR (300 MHz, DMSO-d_6) δ 8.60 (d, *J* = 5.3 Hz, 1H), 8.33 (s, 2H), 7.90 (d, *J* = 7.1 Hz, 1H), 7.34 (s, 1H), 7.24 (d, *J* = 1.9 Hz, 1H), 7.15 – 6.98 (m, 2H), 6.82 (s, 1H), 4.48 (d, *J* = 12.3 Hz, 2H), 3.50 (s, 3H), 3.19 – 3.04 (m, 2H), 2.44 (dd, *J* = 10.2, 5.4 Hz, 1H), 1.88 (dd, *J* = 11.3, 7.7 Hz, 4H).

^{13}C NMR (151 MHz, DMSO-d_6) δ 176.61, 162.61, 154.36, 152.32, 149.60, 148.89, 144.83, 140.59, 139.09, 137.58, 121.85, 116.77, 109.78, 103.17, 50.37, 42.00, 36.99, 28.83.

Compound 98

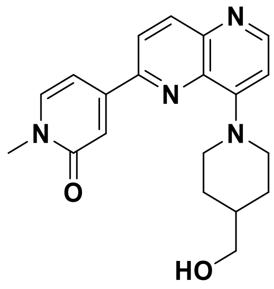

To a solution of 4-(8-chloro-1,5-naphthyridin-2-yl)-1-methylpyridin-2-one (70.mg, 0.26mmol) in *N*,*N*-dimethylacetamide (4mL) was added cesium carbonate (251.83mg, 0.77mmol), followed by the addition of Piperidin-4-ylmethanol (0.12mL, 1.03mmol). The resultant mixture was heated at 110°C in a sealed tube for 18 h. After this time, LC-MS showed formation of the desired compound. The reaction mixture was cooled down and diluted with DCM, filtered, and adsorbed on silica. The compound was purified on ISCO CombiFlash column, using 12g Silicycle and eluting with 90% DCM/10% MeOH. The compound fractions were combined and concentrated in vacuo to give the yellowish product which was purified further by recrystallisation to afford 4-[8-[4-(hydroxymethyl)piperidin-1-yl]-1,5-naphthyridin-2-yl]-1-methylpyridin-2-one (15.11mg,0.0431mmol, 16.737% yield) as a pale-yellow solid compound.

LC-MS: t_R = 0.593 min (method 1, purity 100%); *m/z* = 351.1 $[M+H]^+$ (anal calcd. for $C_{20}H_{22}N_4O_2$: m/z = 350.2).

^{1}H NMR (300 MHz, DMSO-d_6) δ 8.58 (d, *J* = 5.3 Hz, 1H), 8.31 (s, 2H), 7.88 (d, *J* = 7.1 Hz, 1H), 7.23 (d, *J* = 1.9 Hz, 1H), 7.05 (dd, *J* = 7.1, 2.0 Hz, 1H), 7.00 (d, *J* = 5.4 Hz, 1H), 4.57 – 4.39 (m, 3H), 3.50 (s, 3H), 3.14 – 2.94 (m, 2H), 1.87 (dd, *J* = 13.2, 3.6 Hz, 2H), 1.80 – 1.64 (m, 1H), 1.47 (qd, *J* = 12.1, 3.8 Hz, 2H). *Note: some peaks are overlapping with the solvent-water.*

^{1}H NMR (300 MHz, DMSO-d_6 + TFA) δ 8.54 (t, *J* = 8.1 Hz, 2H), 8.38 (d, *J* = 8.9 Hz, 1H), 7.90 (d, *J* = 7.1 Hz, 1H), 7.29 (d, *J* = 7.4 Hz, 1H), 7.20 (d, *J* = 1.9 Hz, 1H), 6.92 (dd, *J* = 7.1, 2.0 Hz, 1H), 3.65 – 3.54 (m, 2H), 3.51 (s, 3H), 3.36 (d, *J* = 5.3 Hz, 2H), 1.96 (dq, *J* = 9.6, 4.1 Hz, 3H), 1.62 – 1.39 (m, 2H).

Compound 99

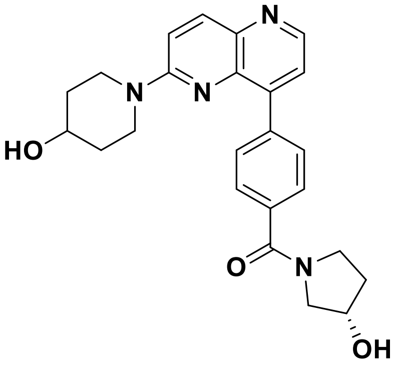

To a 7mL reaction vial was added [1,1′-Bis(diphenylphosphino)ferrocene]dichloropalladium(II) (27.82mg, 0.04mmol), 1-(8-chloro-1,5-naphthyridin-2-yl)piperidin-4-ol (100.mg, 0.38mmol) and [4-[(3S)-3-hydroxypyrrolidine-1-carbonyl]phenyl]boronic acid (106.95mg, 0.46mmol). To this was added degassed 1,4-dioxane (2mL) follwed by degassed water (0.2mL). The reaction mixture was degassed for a further 5 minutes before being heated to 100°C for 18 h on the shaker plate. LCMS confirmed that the reaction had gone to completion and that the desired product had formed.

The mixture was cooled to room temperature, filtered through a pad of celite and the filtrate was adsorbed onto silica gel. Purification was carried out using a Teledyne ISCO CombiFlash system eluting a gradient of MeoH in DCM (0 - 10%) on a 12g Silica column. The product elutes at about 12% MeOH/DCM after some time. The fractions containing the desired product were combined and the solvent removed under reduced pressure and the product dried under high vacuum.

LC-MS: t_R = 0.658 min (method 1, purity 97%); *m/z* = 419.2 $[M+H]^+$ (anal calcd. for $C_{24}H_{26}N_4O_3$: m/z = 418.2).

^{1}H NMR (300 MHz, DMSO-d_6) δ 8.62 (d, *J* = 4.5 Hz, 1H), 8.09 (d, *J* = 9.4 Hz, 1H), 7.92 (d, *J* = 7.9 Hz, 2H), 7.67 – 7.57 (m, 3H), 7.51 (d, *J* = 9.5 Hz, 1H), 4.97 (d, *J* = 20.7 Hz, 1H), 4.69 (s, 1H), 4.31 (d, *J* = 26.2 Hz, 1H), 4.12 (d, *J* = 13.2 Hz, 2H), 3.82 – 3.37 (m, 6H), 3.23 (s, 1H), 1.99 – 1.75 (m, 4H), 1.38 (d, *J* = 10.9 Hz, 2H).

^{13}C NMR (101 MHz, DMSO-d_6) δ 168.19, 156.15, 145.45, 141.75, 140.05, 139.99, 138.44, 136.29, 129.89, 126.57, 126.49, 123.65, 113.26, 69.37, 67.98, 65.94, 56.99, 54.31, 46.79, 44.07, 42.47, 34.33, 33.80, 32.14.

Compound 100

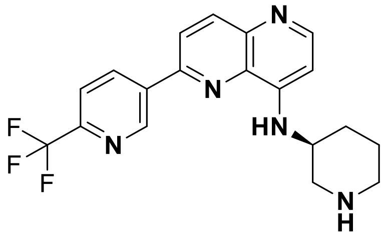

Hydrogen chloride solution (10.mL, 40. mmol) was added to a flask containing tert-butyl (3S)-3-[[6-[6-(trifluoromethyl)pyridin-3-yl]-1,5-naphthyridin-4-yl]amino]piperidine-1-carboxylate (574.mg, 1.21 mmol). The reaction mixture was then left to stir at room

temperature overnight (18 h), LCMS indicating full conversion of the starting material to the desired product after this time. The reaction mixture was concentrated under reduced pressure and the resulting residue diluted with 15mL 10% MeOH in DCM. Amberlyst 21 was added and the suspension stirred for 2 h to neutralize to pH ~ 7. The Amberlyst was filtered off and the filtrate concentrated under reduced pressure. The residue was adsorbed onto isolute and purified using a Teledyne ISCO CombiFlash system eluting a reverse phase solvent gradient of Methanol in 0.1% TFA/Water on a 12g C18 column. The fractions containing the desired product were combined and the solvent removed under reduced pressure (then freeze dried overnight). The residue was taken up in 10mL 10% MeOH/DCM and a small scoop of Amberlyst was added to neutralise the excess TFA. The Amberlyst was filtered off and the filtrate concentrated to dryness to afford 155 mg of the desired product in low purity. The material was then repurified using a Teledyne ISCO CombiFlash system eluting a gradient of 0-10% NH_3(0.5M)/MeOH in DCM on a 12g Silica column to give 140 mg of the desired product in ~90% purity. Finally, 50 mg of the impure product was purified by preparative TLC, developing with 2% NH_3(0.5M)/MeOH solution (multiple times), to afford N-[(3S)-piperidin-3-yl]-6-[6-(trifluoromethyl)pyridin-3-yl]-1,5-naphthyridin-4-amine (31 mg, 0.0830 mmol, 6.85 %) as a yellow solid for submission.

LC-MS: t_R = 0.597 min (method 1, purity 100%); m/z = 374.1 $[M + H]^+$ (anal. calcd. For $C_{19}H_{18}F_3N_5$: m/z = 373.1).

^{1}H NMR (300 MHz, DMSO-d_6) δ 9.76 (d, J = 2.2 Hz, 1H), 8.96 (dd, J = 8.2, 2.2 Hz, 1H), 8.49 (d, J = 5.3 Hz, 1H), 8.45 (d, J = 8.8 Hz, 1H), 8.33 (d, J = 8.8 Hz, 1H), 8.07 (d, J = 8.2 Hz, 1H), 7.33 (d, J = 8.7 Hz, 1H), 6.77 (d, J = 5.5 Hz, 1H), 3.82 – 3.69 (m, 1H), 3.17 (dd, J = 11.8, 3.6 Hz, 1H), 2.99 – 2.87 (m, 1H), 2.83 – 2.72 (m, 1H), 2.72 – 2.60 (m, 1H), 2.04 – 1.91 (m, 1H), 1.85 – 1.68 (m, 2H), 1.67 – 1.52 (m, 1H). *NH not visibe due to D_2O exchange.

^{13}C NMR (151 MHz, DMSO-d_6) δ 152.35, 149.13, 148.88, 146.52 (q, J = 34.0 Hz), 142.77, 138.19, 136.54, 136.48, 134.50, 122.65, 122.34, 120.83, 120.76, 100.67, 49.71, 47.98, 45.35, 29.20, 23.80.

Compound 101

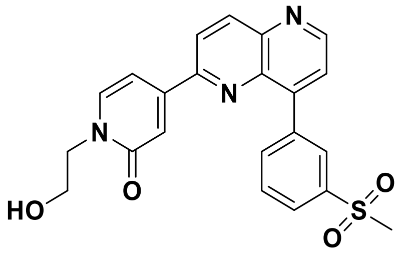

To a microwave tube, was added [8-(3-methylsulfonylphenyl)-1,5-naphthyridin-2-yl] 4-methylbenzenesulfonate (150.mg, 0.33mmol) , [1-(2-hydroxyethyl)-2-oxopyridin-4-yl]boronic acid (120.77mg, 0.66mmol) , [1,1′-

Bis(diphenylphosphino)ferrocene]dichloropalladium(II) (24.21mg, 0.03mmol) and Potassium phosphate tribasic (119.09mg, 0.56mmol) , followed by .1,4-dioxane (5mL) .The resultant reaction mixture was then degassed for few minutes before the addition of water (1mL) . The reaction vessel was then placed in a microwave for 30 minutes , 120°C and 200 W (dynamic mode). When done, LC-MS monitored, the reaction was cooled to room temperature, diluted with DCM and filtered through celite-bed. The crude mixture was purified on Teledyne ISCO CombiFlash system eluting with 90%EtOAc/10% MeOH on a 12g silicycle. The product fractions were combined and concentrated *in vacuo*, recrystallised to afford 1-(2-hydroxyethyl)-4-[8-(3-methylsulfonylphenyl)-1,5-naphthyridin-2-yl]pyridin-2-one (64.45mg,0.1529mmol, 46.34% yield) as an off white solid.

LC-MS: t_R = 0.863 min (method 1, purity 100%); m/z = 422.1 $[M + H]^+$ (anal. calcd. For $C_{22}H_{19}N_3O_4S$: m/z = 421.1).

^1H NMR (300 MHz, DMSO-d_6) δ 9.15 (d, J = 4.4 Hz, 1H), 8.62 (d, J = 8.8 Hz, 1H), 8.58 – 8.45 (m, 2H), 8.28 – 8.20 (m, 1H), 8.11 (dt, J = 8.1, 1.3 Hz, 1H), 8.01 (d, J = 4.5 Hz, 1H), 7.89 (t, J = 7.8 Hz, 1H), 7.70 (d, J = 7.1 Hz, 1H), 7.20 (dd, J = 15.5, 1.8 Hz, 1H), 7.04 (dd, J = 7.1, 2.1 Hz, 1H), 4.90 (t, J = 5.4 Hz, 1H), 4.00 (t, J = 5.4 Hz, 2H), 3.66 (q, J = 5.4 Hz, 2H).

Compound 102

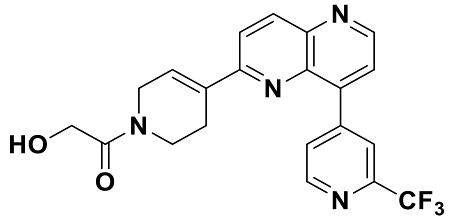

To a solution of 2-(1,2,3,6-tetrahydropyridin-4-yl)-8-[2-(trifluoromethyl)pyridin-4-yl]-1,5-naphthyridine (100.mg, 0.28mmol) in N,N-dimethylformamide (5mL) was added 1-[Bis(dimethylamino)methylene]-1H-1,2,3-triazolo[4,5-b]pyridinium 3-oxid hexafluorophosphate (213.41mg, 0.56mmol), Glycolic acid (25.61mg, 0.34mmol) and stirred for few minutes(5 min). N,N-Diisopropylethylamine (0.15mL, 0.84mmol) was then added to the resulting solution and the reaction mixture was heated at 50°C for 16 h. After this time, LC-MS showed formation of the desired product. After cooling to rt, the reaction mixture was extracted using DCM (2 x 50ml) and water (200 ml). The organic solvent were combined, dried over MgSO4, filtered, and adsorbed on isolute. Purification was achieved on Reverse-Phase CombiFlash ISCO using positive gradient of 0.1%TFA/Methanol on a 12g silicycle. The product fractions were combined and concentrated *in vacuo, excess water was removed on freeze-drier. The product was then stirred in an Amberlyst A21 for 30 minutes (pH 7), filtered and the filtrate was concentrated to dryness to* furnish a light brown solid compound. This product was impure by H NMR, hence second purification ws then obtained by ISCO CombiFlash using 90%DCM -10%MeOH, the product fractions were combined, concentrated and dried in the oven to yield 2-hydroxy-1-[4-[8-[2-(trifluoromethyl)pyridin-4-yl]-1,5-

naphthyridin-2-yl]-3,6-dihydro-2H-pyridin-1-yl]ethanone (12.08mg,0.0292mmol, 10.39% yield) as an off-white solid product.

LC-MS: t_R = 2.425 min (method 2, purity 100%); m/z = 415.1 $[M + H]^+$ (anal. calcd. For $C_{21}H_{17}F_3N_4O_2$: m/z = 414.1).

^1H NMR (400 MHz, DMSO-d_6) δ 9.10 (d, J = 4.7 Hz, 1H), 8.97 (s, 1H), 8.49 (d, J = 10.2 Hz, 2H), 8.30 – 8.17 (m, 2H), 8.05 (s, 1H), 6.99 (d, J = 15.4 Hz, 1H), 4.78 – 4.51 (m, 1H), 4.31 – 4.09 (m, 4H), 3.68 – 3.46 (m, 2H), 2.66 (d, J = 20.9 Hz, 2H).

Compound 103

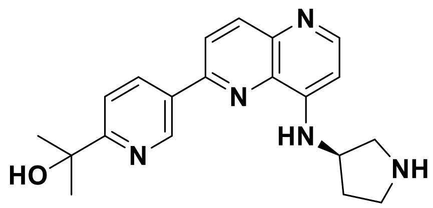

To a solution of tert-butyl rac-(3R)-3-[[6-[6-(2-hydroxypropan-2-yl)pyridin-3-yl]-1,5-naphthyridin-4-yl]amino]pyrrolidine-1-carboxylate (50.mg, 0.11mmol) in methanol (4mL) was added Hydrogen chloride solution (7.52mg, 0.21mmol) The resultant mixture was stirred at room temperature for 18 h. After this time, LC-MS showed formation of the desired compound. To this end, excess of 4M HCl in dioxane was removed *in vacuo*. The residue was re-dissolved with DCM and adsorbed on isolute, purified on Reverse phase using gradient of 0.1%TFA/Methanol. The fractions containing the product were combined, concentrated and Freeze dried to furnish a yellow compound which was then free-based with Amberlyst to give 2-[5-[8-[[rac-(3R)-pyrrolidin-3-yl]amino]-1,5-naphthyridin-2-yl]pyridin-2-yl]propan-2-ol (13.21 mg, 0.0367 mmol, 32.97 %) as a yellow solid compound.

LC-MS: t_R = 0.450 min (method 2, purity 100%); m/z = 350.1 $[M + H]^+$ (anal. calcd. For $C_{20}H_{23}N_5O$: m/z = 349.1).

Compound 104

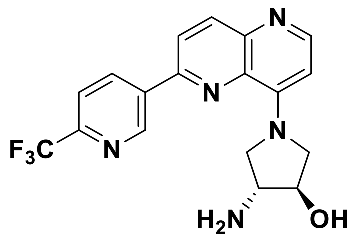

To a solution of benzyl N-[(3R,4R)-4-hydroxy-1-[6-[6-(trifluoromethyl)pyridin-3-yl]-1,5-naphthyridin-4-yl]pyrrolidin-3-yl]carbamate (110.mg, 0.22 mmol) in dichloromethane (5mL) was added Trifluoroacetic acid (10.mL, 130.59 mmol). The resulting reaction mixture was stirred at 60°C for 16 h. After this time, LCMS indicated the appearance of the desired product. TFA was removed on the rotavapor and the residue was then diluted with DCM and adsorbed on isolute.

Purification was achieved on Reverse phase using 0.1%FA/Water-Acetonitrile on a C18 12g silicycle. The product fractions were combined, concentrated and excess water removed on the freeze drier. The product was then stirred in an Amberlyst for 30 minutes (pH 7), filtered and then dried to furnish (3R,4R)-4-amino-1-[6-[6-(trifluoromethyl)pyridin-3-yl]-1,5-naphthyridin-4-yl]pyrrolidin-3-ol (41.35 mg, 0.108 mmol, 50.003 %), as a yellow solid.

LC-MS: t_R = 0.205 min (method 1, purity 98%); *m/z* = 376.1 $[M+H]^+$ (anal calcd. for $C_{18}H_{16}F_3N_5O$: m/z = 375.1).

^1H NMR (300 MHz, DMSO-d_6 + TFA) δ 9.59 (s, 1H), 8.87 (d, *J* = 7.6 Hz, 1H), 8.76 (d, *J* = 9.0 Hz, 1H), 8.64 (d, *J* = 7.1 Hz, 1H), 8.54 (d, *J* = 9.0 Hz, 1H), 8.48 – 8.27 (m, 2H), 8.16 (dd, *J* = 10.8, 8.2 Hz, 1H), 7.07 (dd, *J* = 7.4, 3.4 Hz, 1H), 5.04 – 4.90 (m, 2H), 4.77 – 4.46 (m, 1H), 4.36 – 4.09 (m, 1H), 4.01 – 3.72 (m, 2H).

Compound 105

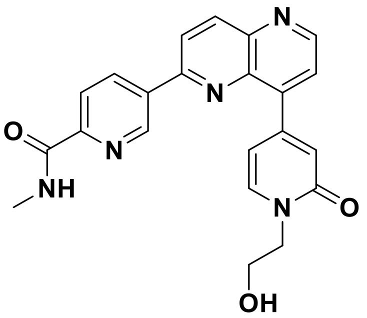

Potassium phosphate tribasic (120.8mg, 0.57 mmol), 5-(8-chloro-1,5-naphthyridin-2-yl)-N-methylpyridine-2-carboxamide (100.mg, 0.33 mmol), [1-(2-hydroxyethyl)-2-oxopyridin-4-yl]boronic acid (73.5mg, 0.4 mmol) and [1,1′-Bis(diphenylphosphino)ferrocene]dichloropalladium(II) (24.56mg, 0.03 mmol) were added to a 100ml rb-flask. To this was added 1,4-dioxane (15 mL) and the reaction was degassed for 5 minutes with N_2. Following this, water (3 mL) that had been degassed for 5 minutes was added and the reaction heated at 120 °C for 18 h until LCMS indicated that the starting material was fully consumed. After this time, the reaction mixture was filtered through a pad of celite and the filtrate adsorbed onto silica gel. Purification was carried out with a Teledyne ISCO CombiFlash system using 90%EtOAc-10%Methanol over 30

min on a 24 g Silica columns. The product elutes at around 90% EtOAc in Methanol. The fractions were combined, concentrated, and triturated using DCM-Pentane. The solid that crushed out was filtered and dried to afford 5-[8-[1-(2-hydroxyethyl)-2-oxopyridin-4-yl]-1,5-naphthyridin-2-yl]-N-methylpyridine-2-carboxamide (53.87 mg, 0.1342 mmol, 40.089 %) as a Pale-Brown solid.

LC-MS: t_R = 0.770 min (method 1, purity 100%); *m/z* = 402.1 $[M+H]^+$ (anal calcd. for $C_{22}H_{19}N_5O_3$: m/z = 401.1).

^{1}H NMR (300 MHz, DMSO-d_6) δ 9.51 – 9.41 (m, 1H), 9.13 – 8.86 (m, 2H), 8.77 (dd, *J* = 8.2, 2.2 Hz, 1H), 8.67 – 8.55 (m, 2H), 8.25 – 8.10 (m, 1H), 7.91 (d, *J* = 4.5 Hz, 1H), 7.79 (d, *J* = 7.0 Hz, 1H), 6.91 (d, *J* = 1.9 Hz, 1H), 6.75 (dd, *J* = 7.0, 2.0 Hz, 1H), 5.04 (t, *J* = 5.3 Hz, 1H), 4.06 (t, *J* = 5.4 Hz, 2H), 3.73 (q, *J* = 5.4 Hz, 2H), 2.85 (d, *J* = 4.9 Hz, 3H).

Compound 106

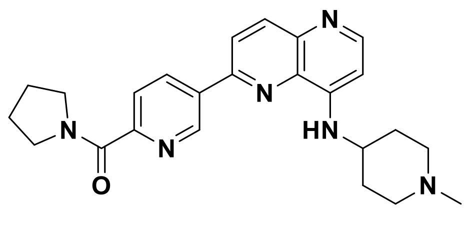

To a solution of [5-(8-chloro-1,5-naphthyridin-2-yl)pyridin-2-yl]-pyrrolidin-1-ylmethanone (437.mg, 1.29 mmol), in 1,4-dioxane (10 mL) was added 4-Amino-1-methylpiperidine (191.48mg, 1.68 mmol), Cesium carbonate (1260.81mg, 3.87 mmol), Palladium(II) acetate (28.96mg, 0.13 mmol) and [1-(2-diphenylphosphanylnaphthalen-1-yl)naphthalen-2-yl]-diphenylphosphane (40.16mg, 0.06 mmol) in that order. The solution was degassed by bubbling nitrogen through the mixture. The reaction was heated at 115 °C until full conversion of the starting reagent (16 h).

The reaction was cooled to room temperature, diluted with ETOAc, and filtered through a pad of celite, washing with more EtOAc followed by MeoH. The crude was then purified by reverse phase (C18) column chromatography eluting a gradient of acetonitrile in water on a Biotage Isolera One Flush Instrument. The desired fractions were concentrated to dryness in vacuo to obtain the product, [5-[8-[(1-methylpiperidin-4-yl)amino]-1,5-naphthyridin-2-yl]pyridin-2-yl]-pyrrolidin-1-ylmethanone (81.96 mg, 0.1968 mmol, 15.255 %), as lemon-green viscous oil, which upon treatment with diethyl ether and pentane mixtures precipitated as solid.

LC-MS: t_R = 0.318 min (method 2, purity 100%); *m/z* = 417.3 $[M+H]^+$ (anal calcd. for $C_{24}H_{28}N_6O$: m/z = 416.2).

^{1}H NMR (600 MHz, DMSO-d_6) δ 9.61 (d, J = 2.3 Hz, 1H), 8.86 (dd, J = 8.2, 2.3 Hz, 1H), 8.45 (d, J = 5.3 Hz, 1H), 8.40 (d, J = 8.8 Hz, 1H), 8.28 (d, J = 8.8 Hz, 1H), 7.88 (d, J = 8.2 Hz, 1H), 7.19 (d, J = 8.4 Hz, 1H), 6.75 (d, J = 5.4 Hz, 1H), 3.74 – 3.67 (m, 2H), 3.61 – 3.52 (m, 3H), 2.85 – 2.79 (m, 2H), 2.23 (s, 3H), 2.16 – 2.08 (m, 2H), 2.00 – 1.94 (m, 2H), 1.93 – 1.85 (m, 4H), 1.84 – 1.75 (m, 2H).

Compound 107

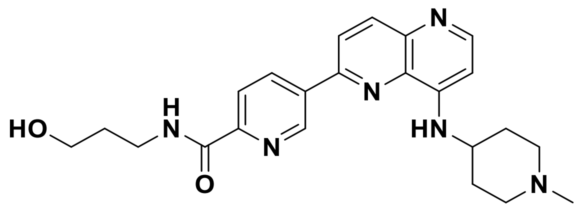

To a solution of 5-(8-chloro-1,5-naphthyridin-2-yl)-N-(3-hydroxypropyl)pyridine-2-carboxamide (60.mg, 0.18 mmol), in 1,4-dioxane (2 mL) was added 4-Amino-1-methylpiperidine (19.99mg, 0.18 mmol), Cesium carbonate (57.03mg, 0.18 mmol), Palladium(II) acetate (39.3mg, 0.18 mmol) and [1-(2-diphenylphosphanylnaphthalen-1-yl)naphthalen-2-yl]-diphenylphosphane (108.99mg, 0.18 mmol) in that order. The solution was degassed by bubbling nitrogen through the mixture. The reaction was heated at 115 °C until full conversion of the starting reagent (5 h).

The reaction was cooled to room temperature, diluted with ETOAc, and filtered through a pad of celite, washing with more EtOAc followed by MeoH. The crude was then purified by reverse phase (C18) column chromatography eluting a gradient of acetonitrile in water on a Biotage Isolera One Flush Instrument. The desired fractions were concentrated to dryness in vacuo to obtain the product, N-(3-hydroxypropyl)-5-[8-[(1-methylpiperidin-4-yl)amino]-1,5-naphthyridin-2-yl]pyridine-2-carboxamide (9 mg, 0.0208 mmol, 11.86 %), as lemon-green viscous oil, which upon treatment with diethyl ether and pentane mixtures precipitated as a yellow solid.

LC-MS: t_R = 0.221 min (method 1, purity 97%); m/z = 421.2 $[M+H]^+$ (anal calcd. for $C_{23}H_{28}N_6O_2$: m/z = 420.2).

^{1}H NMR (300 MHz, DMSO-d_6) δ 9.61 (d, J = 2.2 Hz, 1H), 9.01 – 8.87 (m, 2H), 8.49 – 8.40 (m, 2H), 8.29 (d, J = 8.9 Hz, 1H), 8.17 (d, J = 8.2 Hz, 1H), 7.23 (d, J = 8.4 Hz, 1H), 6.76 (d, J = 5.5 Hz, 1H), 4.66 – 4.58 (m, 1H), 3.61 – 3.48 (m, 4H), 2.86 – 2.75 (m, 2H), 2.22 (s, 3H), 2.17 – 2.04 (m, 2H), 2.03 – 1.87 (m, 2H), 1.87 – 1.65 (m, 4H).

Compound 108

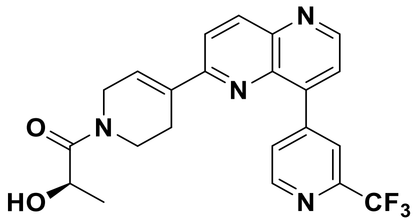

To a solution of 2-(1,2,3,6-tetrahydropyridin-4-yl)-8-[2-(trifluoromethyl)pyridin-4-yl]-1,5-naphthyridine (150.mg, 0.42mmol) in *N*,*N*-dimethylformamide (5mL) was added 1-[Bis(dimethylamino)methylene]-1H-1,2,3-triazolo[4,5-b]pyridinium 3-oxid hexafluorophosphate (320.11mg, 0.84mmol), (2R)-2-hydroxypropanoic acid (45.5mg, 0.51mmol) and stirred for few minutes(5 min). *N*,*N*-Diisopropylethylamine (0.22mL, 1.26mmol) was then added to the resulting solution and the reaction mixture was heated at 50°C for 16 h. After this time, LC-MS showed formation of the desired product. After cooling to rt, the reaction mixture was extracted using DCM (2 x 50ml) and water (200 ml). The organic solvent were combined, dried over $MgSO_4$, filtered, and adsorbed on isolute. Purification was achieved on Reverse-Phase CombiFlash ISCO using positive gradient of 0.1%TFA/Methanol on a 12g silicycle. The product fractions were combined and concentrated *in vacuo, excess water was removed on freeze-drier*. The product was then stirred in an Amberlyst A21 for 30 minutes (pH 7), filtered and the filtrate was concentrated to dryness to furnish (2R)-2-hydroxy-1-[4-[8-[2-(trifluoromethyl)pyridin-4-yl]-1,5-naphthyridin-2-yl]-3,6-dihydro-2H-pyridin-1-yl]propan-1-one (31.7mg,0.0718mmol, 17.051% yield) as a pale-brown solid compound.

LC-MS: t_R = 0.976 min (method 1, purity 97%); *m/z* = 429.1 $[M+H]^+$ (anal calcd. for $C_{22}H_{19}F_3N_4O_2$: m/z = 428.1).

^{1}H NMR (600 MHz, DMSO-d_6) δ 9.04 (d, *J* = 4.4 Hz, 1H), 8.92 (d, *J* = 4.9 Hz, 1H), 8.43 (dd, *J* = 10.0, 6.5 Hz, 2H), 8.24 – 8.09 (m, 2H), 7.99 (d, *J* = 4.4 Hz, 1H), 6.94 (d, *J* = 12.6 Hz, 1H), 4.93 (d, *J* = 6.4 Hz, 1H), 4.54 – 4.12 (m, 2H), 3.82 – 3.39 (m, 2H), 2.71 – 2.52 (m, 2H), 1.18 (dd, *J* = 11.8, 6.6 Hz, 3H). *Note: Traces of ethyl acetate observed.*

Compound 109

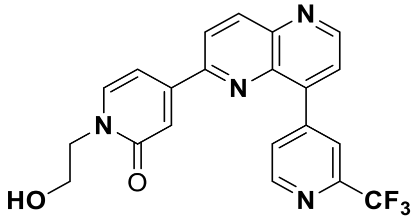

To a microwave tube, was added [8-[2-(trifluoromethyl)pyridin-4-yl]-1,5-naphthyridin-2-yl] 4-methylbenzenesulfonate (110.mg, 0.25mmol), [1-(2-hydroxyethyl)-2-oxopyridin-4-yl]boronic acid (90.37mg, 0.49mmol), [1,1′-Bis(diphenylphosphino)ferrocene]dichloropalladium(II) (18.12mg, 0.02mmol) and Potassium phosphate tribasic (89.12mg, 0.42mmol), followed by .1,4-dioxane (5mL) .The resultant reaction mixture was then degassed for few minutes before the addition of water (1mL). The reaction vessel was then placed in a microwave for 30 minutes, 120°C and 200 W (dynamic mode). When done, LC-MS monitored, the reaction was cooled to room temperature, diluted with DCM and filtered through celite-bed. The crude mixture was purified on reverse phase using 0.1%TFA/Methanol, 12g silicycle. The product fractions were combined and concentrated *in vacuo*, freeze-dried and free-based using Amberlyst to afford 1-(2-hydroxyethyl)-4-[8-[2-(trifluoromethyl)pyridin-4-yl]-1,5-naphthyridin-2-yl]pyridin-2-one (18.88mg,0.0444mmol, 17.98% yield) as an off white solid.

LC-MS: t_R = 0.996 min (method 1, purity 97%); *m/z* = 413.1 $[M+H]^+$ (anal calcd. for $C_{21}H_{15}F_3N_4O_2$: m/z = 412.1).

^{1}H NMR (400 MHz, DMSO-d_6) δ 9.19 (d, *J* = 4.4 Hz, 1H), 9.00 (d, *J* = 4.9 Hz, 1H), 8.64 (s, 1H), 8.53 (d, *J* = 8.9 Hz, 1H), 8.48 (s, 1H), 8.21 (dd, *J* = 5.0, 1.5 Hz, 1H), 8.10 (d, *J* = 4.4 Hz, 1H), 7.74 (d, *J* = 7.1 Hz, 1H), 7.24 (d, *J* = 2.0 Hz, 1H), 6.92 (dd, *J* = 7.1, 2.1 Hz, 1H), 4.93 (s, 1H), 3.99 (t, *J* = 5.4 Hz, 2H), 3.66 (d, *J* = 4.7 Hz, 2H).

Compound 110

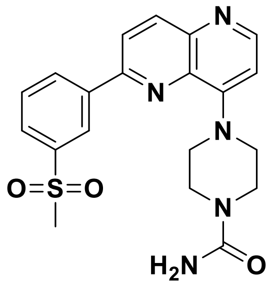

To a 50 mL rbf was added 2 mL DMF and Ammonium acetate (36.82mg, 0.4800mmol) and Triethylamine (0.06mL, 0.4300mmol). After stirring for 2 - 3 minutes, 1,1'-Carbonyldiimidazole (70.41mg, 0.4300mmol) was added and this mixture was stirred for 15 minutes at room temperature. 2-(3-methylsulfonylphenyl)-8-piperazin-1-yl-1,5-naphthyridine (80.mg, 0.2200mmol) was then added and the mixture stirred for a further 18 h at 50°C. After this time, LCMS showed that the desired product had formed. The mixture was extracted with DCM (200 ml) and water (100 ml). The organics were combined, dried over $MgSO_4$, filtered and adsorbed on Isolute. The crude product was purified on Reverse Phase using 0.1%TFA/Water and Methanol. Product fractions were combined, concentrated and excess water was removed on Freeze drier.

The yellowish product that formed was recrystallised using DCM/Hexanes. After filtration and drying of the compound in the vacuum oven ,the desired product 4-[6-(3-methylsulfonylphenyl)-1,5-naphthyridin-4-yl]piperazine-1-carboxamide (30.2mg,0.0734mmol, 30.047% yield) was isolated as a pale-yellow compound.

LC-MS: t_R = 0.275 min (method 1, purity 100%); *m/z* = 412.1 $[M+H]^+$ (anal calcd. for $C_{20}H_{21}N_5O_3S$: m/z = 411.1).

^{1}H NMR (300 MHz, DMSO-d_6**)** δ 8.81 (d, *J* = 1.8 Hz, 1H), 8.62 (dd, *J* = 16.4, 6.5 Hz, 2H), 8.44 (q, *J* = 8.9 Hz, 2H), 8.06 (d, *J* = 7.8 Hz, 1H), 7.87 (t, *J* = 7.8 Hz, 1H), 7.06 (d, *J* = 5.3 Hz, 1H), 6.07 (s, 2H), 3.80 – 3.70 (m, 4H), 3.65 (q, *J* = 4.0, 3.5 Hz, 4H). Note: The CH_3 is ovelapping with the DMSO water peak.

Compound 111

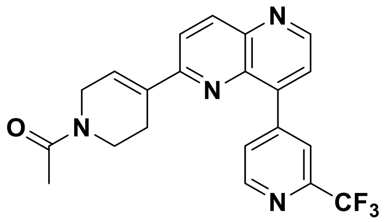

2-(1,2,3,6-tetrahydropyridin-4-yl)-8-[2-(trifluoromethyl)pyridin-4-yl]-1,5-naphthyridine (200.mg, 0.56mmol), dichloromethane (3mL) and Acetic anhydride (0.16mL, 1.68mmol) were added to a rbf, followed by Triethylamine (0.31mL, 2.25mmol). The reaction was stirred at room temperature for 18 hours. LCMS confirmed that the desired product had formed. The reaction mixture was diluted with 5 mL DCM and 8 mL distilled water was added and the mixture stirred for 5 minutes before passing through a phase separator cartridge. The DCM layer was retained, and concentrated under reduced pressure. The resulting residue was adsorbed onto silica and purified using a Teledyne ISCO CombiFlash system eluting a gradient of MeOH in DCM (0 - 10%) on a 12 g Silica column. The fractions containing the desired product were combined and dried under reduced pressure to afford 1-[4-[8-[2-(trifluoromethyl)pyridin-4-yl]-1,5-naphthyridin-2-yl]-3,6-dihydro-2H-pyridin-1-yl]ethanone (119 mg, 0.2987 mmol, 53.22 %) as a pale brown powder.

LC-MS: t_R = 1.096 min (method 1, purity 100%); *m/z* = 399.1 $[M+H]^+$ (anal calcd. for $C_{21}H_{17}F_3N_4O$: m/z = 398.1).

^{1}H NMR (400 MHz, DMSO-d_6) δ 9.08 (d, *J* = 4.5 Hz, 1H), 8.96 (d, *J* = 5.0 Hz, 1H), 8.51 – 8.44 (m, 2H), 8.26 – 8.16 (m, 2H), 8.03 (t, *J* = 4.1 Hz, 1H), 6.98 (d, *J* = 4.7 Hz, 1H), 4.24 (d, *J* = 23.0 Hz, 2H), 3.68 – 3.57 (m, 2H), 2.64 (d, *J* = 36.1 Hz, 2H), 2.06 (d, *J* = 6.7 Hz, 3H).

^{13}C NMR (101 MHz, $CDCl_3$) δ 169.45, 157.59, 150.03, 149.99, 148.09, 147.74, 145.95, 144.07, 143.24, 139.69, 137.42, 135.78, 128.60, 127.47, 126.79, 123.74, 122.96, 121.41, 77.36, 77.04, 76.72, 46.03, 43.28, 42.38, 38.18, 26.06, 21.50

References

(1) Shen, Z.; Ratia, K.; Cooper, L.; Kong, D.; Lee, H.; Kwon, Y.; Li, Y.; Alqarni, S.; Huang, F.; Dubrovskyi, O.; Rong, L.; Thatcher, G. R. J.; Xiong, R. Design of SARS-CoV-2 PLpro Inhibitors for COVID-19 Antiviral Therapy Leveraging Binding Cooperativity. *J Med Chem* **2022**, *65* (4), 2940–2955. https://doi.org/10.1021/acs.jmedchem.1c01307.

(2) Fourches, D.; Muratov, E.; Tropsha, A. Trust, but Verify II: A Practical Guide to Chemogenomics Data Curation. *Journal of Chemical Information and Modeling* **2016**, *56* (7), 1243–1252. https://doi.org/10.1021/acs.jcim.6b00129.

(3) Fourches, D.; Muratov, E.; Tropsha, A. Trust, but Verify: On the Importance of Chemical Structure Curation in Cheminformatics and QSAR Modeling Research. *Journal of chemical information and modeling* **2010**, *50* (7), 1189–1204. https://doi.org/10.1021/ci100176x.

(4) O'Boyle, N. M.; Banck, M.; James, C. A.; Morley, C.; Vandermeersch, T.; Hutchison, G. R. Open Babel: An Open Chemical Toolbox. *Journal of Cheminformatics* **2011**, *3* (1), 33. https://doi.org/10.1186/1758-2946-3-33.

(5) Hawkins, P. C. D.; Skillman, A. G.; Warren, G. L.; Ellingson, B. A.; Stahl, M. T. Conformer Generation with OMEGA: Algorithm and Validation Using High Quality Structures from the Protein Databank and Cambridge Structural Database. *Journal of Chemical Information and Modeling* **2010**, *50* (4), 572–584. https://doi.org/10.1021/ci100031x.

(6) Jakalian, A.; Bush, B. L.; Jack, D. B.; Bayly, C. I. Fast, Efficient Generation of High-Quality Atomic Charges. AM1-BCC Model: I. Method. *Journal of Computational Chemistry* **2000**, *21* (2), 132–146. https://doi.org/10.1002/(SICI)1096-987X(20000130)21:2<132::AID-JCC5>3.0.CO;2-P.

(7) Jakalian, A.; Jack, D. B.; Bayly, C. I. Fast, Efficient Generation of High-quality Atomic Charges. AM1-BCC Model: II. Parameterization and Validation. *Journal of Computational Chemistry* **2002**, *23* (16), 1623–1641. https://doi.org/10.1002/jcc.10128.

(8) QUACPAC 2.0.1.2. OpenEye, Cadence Molecular Sciences, Santa Fe, NM. Http://Www.Eyesopen.Com.

(9) ROCS 3.4.2.1. OpenEye, Cadence Molecular Sciences, Santa Fe, NM. Http://Www.Eyesopen.Com.

(10) Hawkins, P. C. D.; Skillman, A. G.; Nicholls, A. Comparison of Shape-Matching and Docking as Virtual Screening Tools. *Journal of Medicinal Chemistry* **2007**, *50* (1), 74–82. https://doi.org/10.1021/jm0603365.

(11) Braga, R. C.; Andrade, C. H. Assessing the Performance of 3D Pharmacophore Models in Virtual Screening: How Good Are They? *Current Topics in Medicinal Chemistry* **2013**, *13* (9), 1127–1138. https://doi.org/10.2174/1568026611313090010.

(12) Neves, B. J.; Dantas, R. F.; Senger, M. R.; Melo-Filho, C. C.; Valente, W. C. G.; De Almeida, A. C. M.; Rezende-Neto, J. M.; Lima, E. F. C.; Paveley, R.; Furnham, N.; Muratov, E.; Kamentsky, L.; Carpenter, A. E.; Braga, R. C.; Silva-Junior, F. P.; Andrade, C. H. Discovery of New Anti-Schistosomal Hits by Integration of QSAR-Based Virtual Screening and High Content Screening.

Journal of Medicinal Chemistry **2016**, *59* (15), 7075–7088. https://doi.org/10.1021/acs.jmedchem.5b02038.

(13) Schrödinger Release 2024-2: Protein Preparation Wizard; Epik, Schrödinger, LLC, New York, NY, 2024; Impact, Schrödinger, LLC, New York, NY; Prime, Schrödinger, LLC, New York, NY, 2024. LCC: New York, NY, USA 2015.

(14) Madhavi Sastry, G.; Adzhigirey, M.; Day, T.; Annabhimoju, R.; Sherman, W. Protein and Ligand Preparation: Parameters, Protocols, and Influence on Virtual Screening Enrichments. *Journal of Computer-Aided Molecular Design* **2013**, *27* (3), 221–234. https://doi.org/10.1007/s10822-013-9644-8.

(15) Schrödinger Release 2021-4: Maestro, Schrödinger, LLC, New York, NY, 2024.

(16) Shelley, J. C.; Cholleti, A.; Frye, L. L.; Greenwood, J. R.; Timlin, M. R.; Uchimaya, M. Epik: A Software Program for PK(a) Prediction and Protonation State Generation for Drug-like Molecules. *Journal of computer-aided molecular design* **2007**, *21* (12), 681–691. https://doi.org/10.1007/s10822-007-9133-z.

(17) Schrödinger Release 2024-1: Epik, Schrödinger, 2024, LLC New York, NY, USA. LLC, New York, NY.

(18) Schrödinger Release 2024-2: Force Fields, Schrödinger, LLC, New York, NY, 2024.

(19) Shivakumar, D.; Williams, J.; Wu, Y.; Damm, W.; Shelley, J.; Sherman, W. Prediction of Absolute Solvation Free Energies Using Molecular Dynamics Free Energy Perturbation and the OPLS Force Field. *Journal of Chemical Theory and Computation* **2010**, *6* (5), 1509–1519. https://doi.org/10.1021/ct900587b.

(20) Schrödinger. Schrödinger Release 2024-2: LigPrep, Schrödinger, LLC, New York, NY, 2024. *Schrödinger Release 2018-2*. LLC, New York, NY, 2024 2024.

(21) Schrödinger, L. Schrödinger Release 2024-2: Glide, Schrödinger, LLC, New York, NY, 2024. New York, NY 2024.

(22) Fu, Z.; Huang, B.; Tang, J.; Liu, S.; Liu, M.; Ye, Y.; Liu, Z.; Xiong, Y.; Zhu, W.; Cao, D.; Li, J.; Niu, X.; Zhou, H.; Zhao, Y. J.; Zhang, G.; Huang, H. The Complex Structure of GRL0617 and SARS-CoV-2 PLpro Reveals a Hot Spot for Antiviral Drug Discovery. *Nat Commun* **2021**, *12* (1), 1–12. https://doi.org/10.1038/s41467-020-20718-8.

(23) Halgren, T. A.; Murphy, R. B.; Friesner, R. A.; Beard, H. S.; Frye, L. L.; Pollard, W. T.; Banks, J. L. Glide: A New Approach for Rapid, Accurate Docking and Scoring. 2. Enrichment Factors in Database Screening. *Journal of Medicinal Chemistry* **2004**, *47* (7), 1750–1759. https://doi.org/10.1021/jm030644s.

(24) Friesner, R. a; Banks, J. L.; Murphy, R. B.; Halgren, T. a; Klicic, J. J.; Mainz, D. T.; Repasky, M. P.; Knoll, E. H.; Shelley, M.; Perry, J. K.; Shaw, D. E.; Francis, P.; Shenkin, P. S. Glide: A New Approach for Rapid, Accurate Docking and Scoring. 1. Method and Assessment of Docking Accuracy. *Journal of medicinal chemistry* **2004**, *47* (7), 1739–1749. https://doi.org/10.1021/jm0306430.

(25) Berthold, M. R.; Cebron, N.; Dill, F.; Gabriel, T. R.; Kötter, T.; Meinl, T.; Ohl, P.; Sieb, C.; Thiel, K.; Wiswedel, B. KNIME: The Konstanz Information Miner. In *Studies in Classification, Data Analysis, and Knowledge Organization*; Springer, Berlin, Heidelberg, 2008.

(26) Fillbrunn, A.; Dietz, C.; Pfeuffer, J.; Rahn, R.; Landrum, G. A.; Berthold, M. R. KNIME for Reproducible Cross-Domain Analysis of Life Science Data. *Journal of Biotechnology* **2017**, *261*, 149–156. https://doi.org/10.1016/j.jbiotec.2017.07.028.

(27) Neves, B.; Moreira-Filho, J.; Silva, A.; Borba, J.; Mottin, M.; Alves, V.; Braga, R.; Muratov, E.; Andrade, C. Automated Framework for Developing Predictive Machine Learning Models for Data-Driven Drug Discovery. *Journal of the Brazilian Chemical Society* **2021**. https://doi.org/10.21577/0103-5053.20200160.

(28) Sander, T.; Freyss, J.; Von Korff, M.; Rufener, C. DataWarrior: An Open-Source Program for Chemistry Aware Data Visualization and Analysis. *Journal of Chemical Information and Modeling* **2015**. https://doi.org/10.1021/ci500588j.

(29) Salentin, S.; Schreiber, S.; Haupt, V. J.; Adasme, M. F.; Schroeder, M. PLIP: Fully Automated Protein–Ligand Interaction Profiler. *Nucleic Acids Research* **2015**, *43* (W1), W443–W447. https://doi.org/10.1093/nar/gkv315.

(30) Kandepedu, N.; Gonzàlez Cabrera, D.; Eedubilli, S.; Taylor, D.; Brunschwig, C.; Gibhard, L.; Njoroge, M.; Lawrence, N.; Paquet, T.; Eyermann, C. J.; Spangenberg, T.; Basarab, G. S.; Street, L. J.; Chibale, K. Identification, Characterization, and Optimization of 2,8-Disubstituted-1,5-Naphthyridines as Novel Plasmodium Falciparum Phosphatidylinositol-4-Kinase Inhibitors with in Vivo Efficacy in a Humanized Mouse Model of Malaria. *Journal of Medicinal Chemistry* **2018**, *61* (13), 5692–5703. https://doi.org/10.1021/acs.jmedchem.8b00648.

(31) Dziwornu, G. A.; Seanego, D.; Fienberg, S.; Clements, M.; Ferreira, J.; Sypu, V. S.; Samanta, S.; Bhana, A. D.; Korkor, C. M.; Garnie, L. F.; Teixeira, N.; Wicht, K. J.; Taylor, D.; Olckers, R.; Njoroge, M.; Gibhard, L.; Salomane, N.; Wittlin, S.; Mahato, R.; Chakraborty, A.; Sevilleno, N.; Coyle, R.; Lee, M. C. S.; Godoy, L. C.; Pasaje, C. F.; Niles, J. C.; Reader, J.; van der Watt, M.; Birkholtz, L.-M.; Bolscher, J. M.; de Bruijni, M. H. C.; Coulson, L. B.; Basarab, G. S.; Ghorpade, S. R.; Chibale, K. 2,8-Disubstituted-1,5-Naphthyridines as Dual Inhibitors of Plasmodium Falciparum Phosphatidylinositol-4-Kinase and Hemozoin Formation with In Vivo Efficacy. *Journal of Medicinal Chemistry* **2024**. https://doi.org/10.1021/acs.jmedchem.4c01154.

(32) Andrade, M. A.; Mottin, M.; Sousa, B. K. de P.; Barbosa, J. A. R. G.; dos Santos Azevedo, C.; Lasse Silva, C.; Gonçalves de Andrade, M.; Motta, F. N.; Maulay-Bailly, C.; Amand, S.; Santana, J. M. de; Horta Andrade, C.; Grellier, P.; Bastos, I. M. D. Identification of Novel Zika Virus NS3 Protease Inhibitors with Different Inhibition Modes by Integrative Experimental and Computational Approaches. *Biochimie* **2023**, *212*, 143–152. https://doi.org/10.1016/j.biochi.2023.04.004.

(33) Cornish-Bowden, A. *Fundamentals of Enzyme Kinetics*, 4th editio.; Portland Press: Lodon, 1996.

(34) Freire, M. C. L. C.; Noske, G. D.; Bitencourt, N. V.; Sanches, P. R. S.; Santos-Filho, N. A.; Gawriljuk, V. O.; de Souza, E. P.; Nogueira, V. H. R.; de Godoy, M. O.; Nakamura, A. M.; Fernandes, R. S.; Godoy, A. S.; Juliano, M. A.; Peres, B. M.; Barbosa, C. G.; Moraes, C. B.; Freitas-Junior, L. H. G.; Cilli, E. M.; Guido, R. V. C.; Oliva, G. Non-Toxic Dimeric Peptides Derived from the Bothropstoxin-I Are Potent SARS-CoV-2 and Papain-like Protease Inhibitors. *Molecules* **2021**, *26* (16), 4896. https://doi.org/10.3390/molecules26164896.

(35) Grøftehauge, M. K.; Hajizadeh, N. R.; Swann, M. J.; Pohl, E. Protein–Ligand Interactions Investigated by Thermal Shift Assays (TSA) and Dual Polarization Interferometry (DPI). *Acta*

Crystallographica Section D Biological Crystallography **2015**, *71* (1), 36–44. https://doi.org/10.1107/S1399004714016617.
